# The importance of accounting method and sampling depth to estimate changes in soil carbon stocks

**DOI:** 10.1186/s13021-024-00249-1

**Published:** 2024-01-26

**Authors:** Anna M. Raffeld, Mark A. Bradford, Randall D. Jackson, Daniel Rath, Gregg R. Sanford, Nicole Tautges, Emily E. Oldfield

**Affiliations:** 1 Environmental Defense Fund, 555 12th Street, Suite 400, Washington, DC 20004 USA; 2 The Forest School, Yale School of the Environment, Yale University, 360 Prospect St., New Haven, CT 06511 USA; 3 Department of Plant and Agroecosystem Sciences, University of Wisconsin-Madison, 1575 Linden Drive, Madison, WI 53706 USA; 4 Natural Resources Defense Council, 1152 15th St NW, Washington, DC 20005 USA; 5 Michael Fields Agricultural Institute, East Troy, WI PO Box 990, 53120 USA

**Keywords:** Bulk density, Carbon accounting, Carbon stocks, Fixed depth, Equivalent soil mass, Carbon markets, Soil carbon

## Abstract

**Background:**

As interest in the voluntary soil carbon market surges, carbon registries have been developing new soil carbon measurement, reporting, and verification (MRV) protocols. These protocols are inconsistent in their approaches to measuring soil organic carbon (SOC). Two areas of concern include the type of SOC stock accounting method (fixed-depth (FD) vs. equivalent soil mass (ESM)) and sampling depth requirement. Despite evidence that fixed-depth measurements can result in error because of changes in soil bulk density and that sampling to 30 cm neglects a significant portion of the soil profile’s SOC stock, most MRV protocols do not specify which sampling method to use and only require sampling to 30 cm. Using data from UC Davis’s Century Experiment (“Century”) and UW Madison’s Wisconsin Integrated Cropping Systems Trial (WICST), we quantify differences in SOC stock changes estimated by FD and ESM over 20 years, investigate how sampling at-depth (> 30 cm) affects SOC stock change estimates, and estimate how crediting outcomes taking an empirical sampling-only crediting approach differ when stocks are calculated using ESM or FD at different depths.

**Results:**

We find that FD and ESM estimates of stock change can differ by over 100 percent and that, as expected, much of this difference is associated with changes in bulk density in surface soils (e.g., *r* = 0.90 for Century maize treatments). This led to substantial differences in crediting outcomes between ESM and FD-based stocks, although many treatments did not receive credits due to declines in SOC stocks over time. While increased variability of soils at depth makes it challenging to accurately quantify stocks across the profile, sampling to 60 cm can capture changes in bulk density, potential SOC redistribution, and a larger proportion of the overall SOC stock.

**Conclusions:**

ESM accounting and sampling to 60 cm (using multiple depth increments) should be considered best practice when quantifying change in SOC stocks in annual, row crop agroecosystems. For carbon markets, the cost of achieving an accurate estimate of SOC stocks that reflect management impacts on soils at-depth should be reflected in the price of carbon credits.

**Supplementary Information:**

The online version contains supplementary material available at 10.1186/s13021-024-00249-1.

## Background

The voluntary carbon market for agricultural soil carbon sequestration is expanding at a rapid pace and a number of monitoring, reporting, and verification (MRV) protocols intended to generate verified credits have been published in the past three years [1]. The voluntary carbon market is designed to financially incentivize farmers to adopt climate-smart agricultural practices (e.g., reduced and no-tillage, cover cropping) that may draw down atmospheric CO_2_ to help mitigate climate change. One credit, therefore, is equivalent to 1 tonne (Mg) of CO_2_e. With credits potentially sold and used as offsets for industrial emissions, the stakes of the voluntary carbon market are high, as the success of climate mitigation efforts depends on the accuracy of these credits. Protocols should therefore incorporate best practices for measurement and estimation of soil organic carbon (SOC) and other greenhouse gases (e.g., N_2_O and CH_4_) to ensure that credits result in real climate benefits. However, MRV protocols are not currently using adequately robust methods with built-in empirical verification to accurately estimate carbon credits [2, 3].

Empirically detecting change in SOC is a critically important component of carbon crediting. Traditional approaches to measuring and estimating SOC stocks rely on a fixed depth (FD) approach that involves multiplying a specific soil depth increment by its corresponding bulk density and carbon (C) concentration. A major issue with the FD approach is that it does not account or correct for changes in soil bulk density that often occur with shifts in management practices. An increase or decrease in bulk density can change the mass of soil sampled for a specific depth increment (e.g., 0–30 cm). Depending on the direction of bulk density change, carbon accounting on a fixed depth basis could result in either under- or over-accounting of SOC stocks [4]. By contrast, the equivalent soil mass (ESM) approach calculates SOC stocks using the mass of soil for a given reference layer (in this case, the reference layer refers to the initial fixed depth increment) (Additional file 1: Tables S1a–c); this is a measurement that, by definition, does not vary with bulk density. A large body of research has shown the importance of calculating stocks on an ESM basis and the bias introduced by using the FD approach [5–12]; however, since the ESM method can also introduce error, care should be taken to minimize this error by using multiple depth intervals (e.g., 0–15, 15–30, 30–45, 45–60 cm as opposed to 0–30 or 0–60 cm) for stock calculation, and accounting for non-linear rates of change for SOC and bulk density across the soil profile by using cubic spline interpolation [8, 12, 13]. Notably, a synthesis of twelve publicly-available MRV protocols revealed that of the protocols that include a sampling requirement, only three (BCarbon’s Soil Carbon Protocol, Australia’s Carbon Credits Methodology Determination, and VM0042 Methodology for Improved Agricultural Land Management, v2.0) require or suggest calculating SOC stocks using ESM [14].

In addition to the potential bias generated by FD sampling, current MRV protocols typically require sampling to a depth of only 30 cm. In the previously mentioned protocol review, only one protocol required sampling at depths below 30 cm, likely related to the additional time, cost and effort required to collect deep soil samples. These logistical challenges have resulted in a lack of sub-surface soil data [15–17]. Yet, an accurate estimation of SOC stocks should ideally incorporate sampling below surface soils (e.g., > 30 cm). Soils store a large amount of SOC below 30 cm (around 55% of SOC to 1 m can be stored below 30 cm [18]), and this subsurface carbon can be up to thousands of years old, meaning that subsurface soils offer substantial potential for long-term carbon sequestration [19–21]. Further, sampling only surface soils may not capture the redistribution of SOC that can occur across the soil profile under practices such as no-tillage (for example, when SOC becomes concentrated in the surface, but less is incorporated into the plow layer and below than with tillage), calling into question the overall mitigation potential of this practice [5, 10, 22, 23]. There are also large uncertainties associated with the impact of no-tillage (among other practices) on subsurface soils [15, 17]. Accurately capturing and quantifying dynamics of SOC at depth poses the classic signal-to-noise challenge given exceedingly small changes of SOC at depth (e.g., carbon concentrations < 0.05%) against an increasingly variable soil environment.

**Fig. 1:**
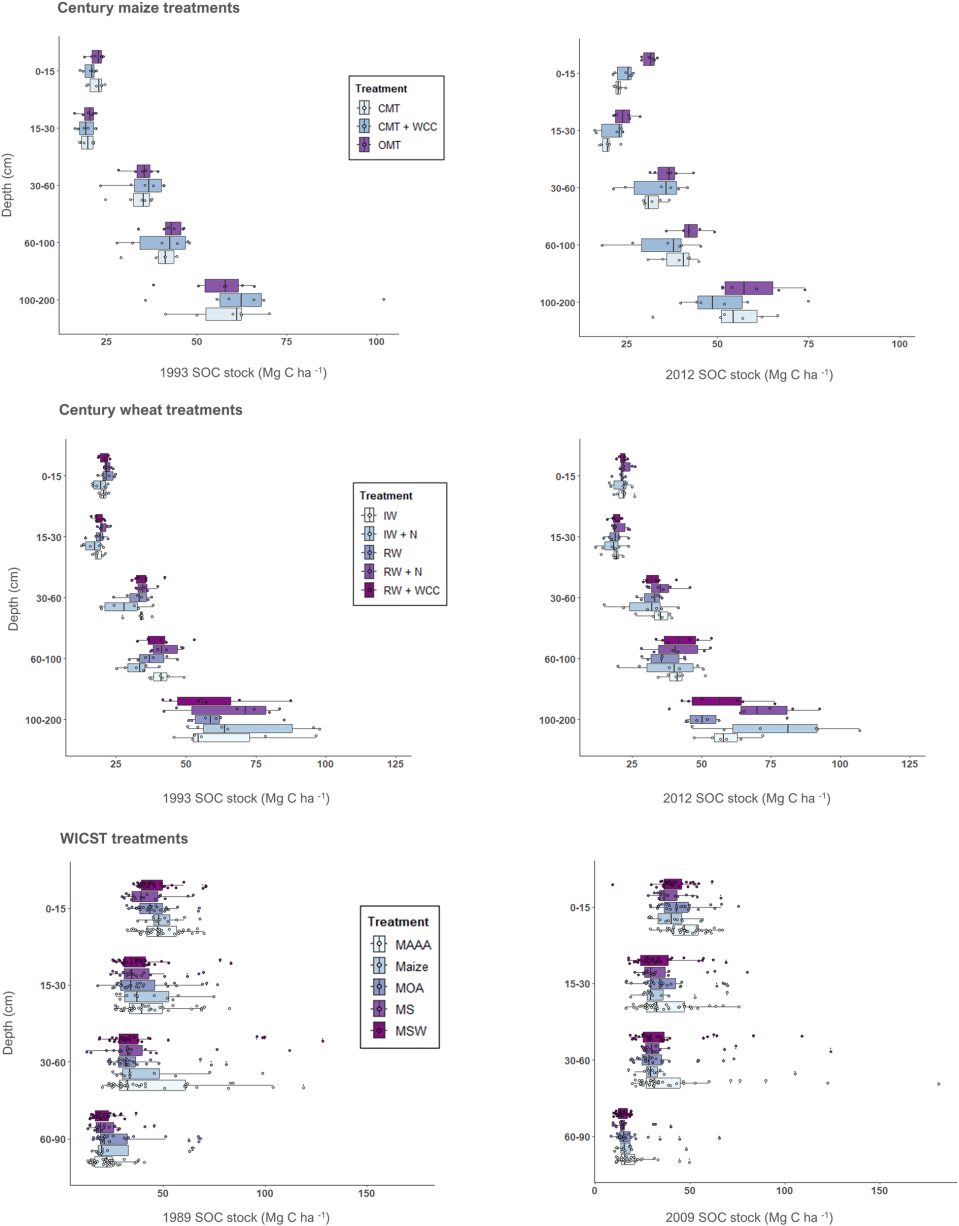
Boxplots of average ESM SOC stock by year, treatment, and depth. Boxplots showing SOC stock (Mg C ha^−1^) distribution by year, treatment, and depth; n = 6 for each Century experiment treatment (both maize and wheat treatments). For the WICST treatments, n = 12 for Maize, n = 18 for MS, n = 33 for MSW, n = 39 for MAAA, and n = 27 for MOA

Given that additional soil MRV protocols are being developed and that consistency is lacking across current protocols, the carbon accounting and sampling methods in these soil MRV protocols must be critically assessed to ensure they accurately reflect changes in SOC [4]. Here, we use experimental data from two long-term cropping systems trials that measured and quantified SOC at-depth: UC Davis’s Century Experiment (“Century”) and UW Madison’s Wisconsin Integrated Cropping Systems Trial (WICST) (see Fig. 1 for initial and final ESM SOC stock estimates across treatments, years and soil layers). These experiments contain a range of treatments, in which soil carbon concentration and bulk density were measured across depth-increments (up to 90 cm at WICST and 200 cm at Century) at two points in time (see Tables 1 and 2 for treatment descriptions and methods for full details). We quantified differences in SOC stock changes estimated by FD and ESM and investigated how sampling at depth (> 30 cm) affected SOC stock change estimates across the entire soil profile. We show that the magnitude and direction of change can vary dramatically depending on the SOC accounting method (ESM vs. FD) and sampling depth. For example, in the Century experiment’s conventional maize—tomato treatment’s (CMT) top 15 cm, there was an average difference of 4.18 Mg C ha^−1^ between ESM and FD estimates of stock change, with FD estimating a decrease and ESM estimating an increase in stock. Finally, to test the implications of number of carbon credits issued, we employ a crediting approach based on empirical soil sampling to compare how the number of credits that would be issued to each Century and WICST cropping system differs depending on the methodology applied (ESM vs. FD). The overarching goal of this analysis was to provide empirical, evidence-based recommendations to support robust and accurate standards for calculating SOC stock changes in soil carbon MRV protocols.

**Table 1:** Century treatment descriptions^a^

Century maize treatments^b^	
CMT	Conventional maize–tomato with synthetic fertilizer, pesticides, and winter fallow
CMT + WCC	A hybrid system with synthetic fertilizer, pesticide, and a winter cover crop (WCC)
OMT	Certified organic maize–tomato with composted poultry manure and WCC
Century wheat treatments^c^	
RW	Rainfed wheat–fallow control with no additional inputs
RW + N	Rainfed wheat–fallow and N fertilizer
RW + WCC	Rainfed wheat–fallow with WCC planted after wheat harvest and terminated before summer fallow
IW	Irrigated wheat–fallow with winter supplemental irrigation and no fertilizer inputs
IW + N	Irrigated wheat–fallow with supplemental irrigation and N fertilizer

^a^Across all Century treatments, disking operations were restricted to 15–20 cm depths, and tillage was conducted to a maximum depth of 25 cm. Soil sampling across all treatments occurred in fall prior to any tillage event

^b^Post-harvest residues for both the maize and tomato rotations were disked into the soil. Tomato seedlings were started in greenhouses and transferred to beds prepared by listing and rolling. WCC were terminated by mowing and incorporated with 2–3 disking operations

^c^Post-harvest wheat residues across all wheat treatments were incorporated by two shallow disking operations in summer. For treatments with fallow, four disking operations occurred after wheat harvest to manage weeds. WCC were terminated with 2–3 disking operations

**Table 2:** WICST treatment descriptions^a^

WICST grain treatments	
Maize	High-external input, continuous maize system
MS	Moderate external input, no-till maize-soybean system
MSW	Organic maize-soybean-wheat followed by cover crop
WICST forage treatments	
MAAA	Maize-alfalfa-alfalfa-alfalfa rotation
MOA	Organic maize-oats/alfalfa/alfalfa rotation

^a^All soil sampling occurred in the fall. Where applicable, this was done after crop harvest but before tillage. Tillage did not exceed 20 cm for all treatments. See Additional file 1: Table S2 for specific tillage information for the WICST treatments

## Results

### Century experiment

#### Maize treatments: carbon concentration and bulk density change from 1993 to 2012

Accounting for changes in bulk density and carbon concentration was important as both likely played a role in the differences between the ESM and FD stock change estimates. In all the Century maize treatments, the majority of the bulk density change occurred in the top 60 cm (up to a 20% change, see Additional file 1: Table S3), with the greatest decreases occuring in the top 30 cm (Additional file 1: Fig. S1). At 0–15 and 15–30 cm, the average decrease in bulk density for all treatments between 1993 and 2012 was greater than 11% and nearly all changes (except for CMT) were significant (see Additional file 1: Tables S3 and S4, and Fig. S1 for all treatments, depths and t test results). At 30–60 cm, treatment CMT + WCC showed a ~ 12% decrease in bulk density of 0.18 g (grams) cm^−3^ (95% CI [−0.28, −0.08]). For all treatments, there were minimal changes in bulk density below 60 cm (Additional file 1: Tables S3, S4).

The effect size—or magnitude of difference—between the 1993 and 2012 carbon concentrations in CMT + WCC and OMT’s 0–15 cm depth increment was large, likely due to the addition of organic matter inputs over time in these treatments (Additional file 1: Table S3): CMT + WCC’s carbon concentration increased by 1.91 g C kg^−1^ (95% CI [0.63, 3.19], *hedges’ g effect size (g)* = *1.79; p* = *0.008*) and OMT’s increased by 4.01 g C kg^−1^ (95% CI [2.90, 5.12], *g* = *4.31; p* = *0.00001*) (Additional file 1: Fig. S2), a 21% and 41% increase, respectively (Additional file 1: Table S3). OMT was the only treatment with a large effect size at depth 15–30 cm (a 22% increase of 1.99 g C kg^−1^; 95% CI [0.45, 3.52], *g* = *1.56, p* = *0.017*) (Additional file 1: Table S3). While OMT saw slight increases in carbon concentrations below 30 cm, these increases were small (less than 0.40 g C kg^−1^) with confidence intervals all overlapping with 0 (Additional file 1: Table S3, Fig. S2). Both CMT and CMT + WCC saw decreases in carbon concentration across 30–200 cm, however, there was a large degree of variability associated with these changes (Additional file 1: Fig. S2). For average bulk density and carbon concentrations see Additional file 1: Table S6, and for statistical output, see Additional file 1: Tables S4 and S5.

#### Maize treatments SOC stock change from 1993 to 2012

**Fig. 2:**
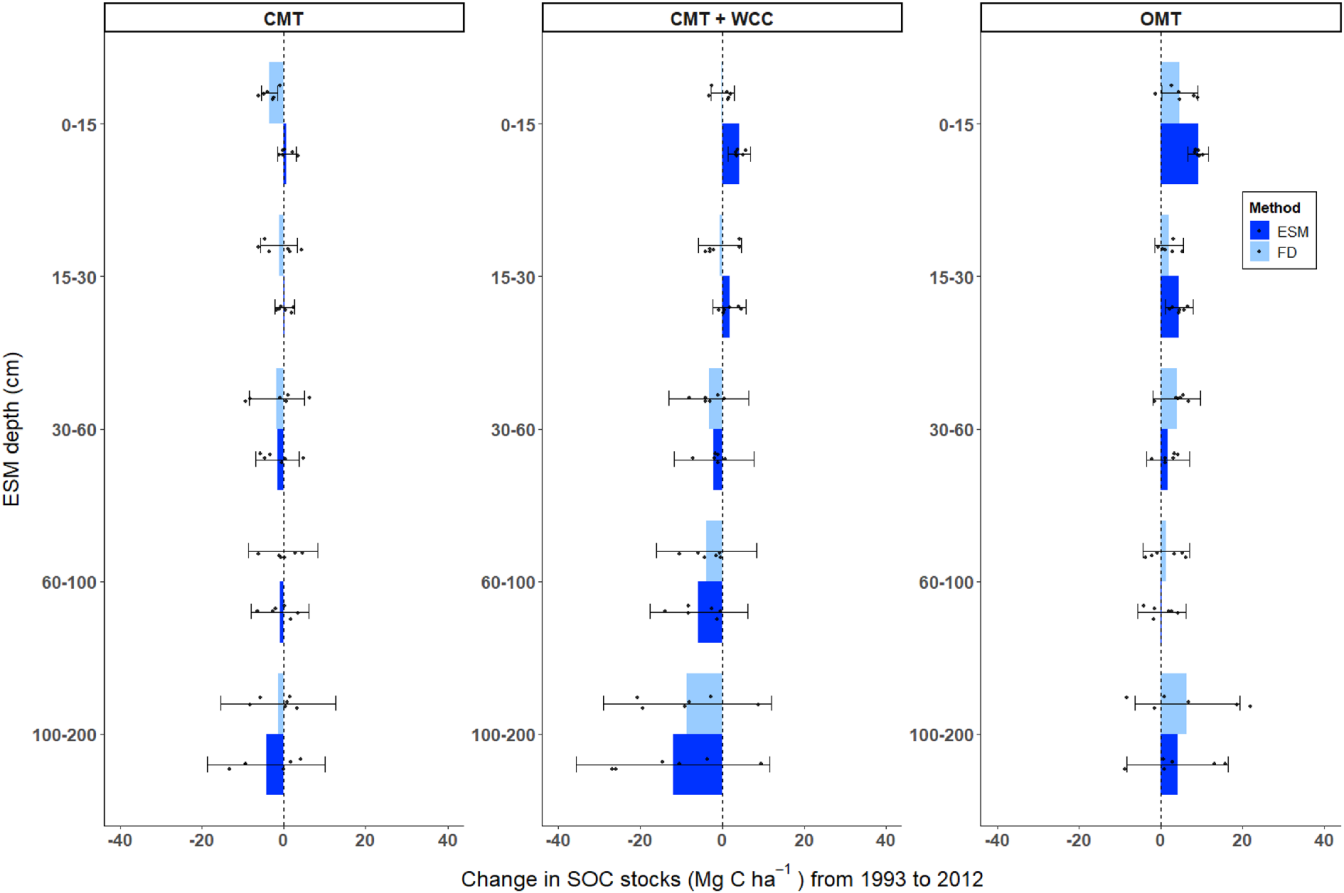
Century maize SOC stock change from 1993 to 2012 as estimated by FD and ESM. Average change from 1993 to 2012 in SOC stocks (Mg C ha^−1^) by soil depth in each maize Century treatment (n = 6 per treatment) as estimated by both FD and ESM, with individual observations represented by black circles. CMT refers to the conventional maize treatment, LMT is conventional maize with winter cover crops, and OMTF is organic maize. We report the results as “ESM depth” intervals [8]. These intervals are the depths represented by the reference soil masses used for the ESM calculation. The reference mass is defined as the mass from the initial fixed depth sample averaged across treatment replicates. Reference masses for each treatment and depth interval can be found in Additional file 1: Table S1a. Error bars represent 95% confidence intervals

For all maize treatments, there was a large magnitude of difference between the stock change estimated by ESM and FD at 0–15 cm. In CMT, ESM accounting estimated a 3% increase in stock of 0.65 Mg C ha^−1^ (95% CI [−1.63, 2.94]) while FD showed a 16% decrease of −3.53 Mg C ha^−1^ (95% CI [−5.45, −1.61]), resulting in an average difference, or FD error, of 4.18 Mg C ha^−1^ (*g* = *2.11; p* = *0.003*) (Table 3, Fig. 2). Although both accounting methods estimated stock increases for CMT + WCC and OMT at 0–15 cm, the ESM change estimates were larger than FD estimates by 4.07 Mg C ha^−1^ for CMT + WCC (*g* = *2.06; p* = *0.006*) and 4.59 Mg C ha^−1^ for OMT (*g* = *1.57; p* = *0.030*) (Table 3, Fig. 2).

**Table 3:** T-test results with confidence intervals for the difference between Century maize ESM and FD mean stock change estimates

Treatment^b^	ESM depth (cm)	Difference in mean between ESM and FD stock change^a^ (Mg C ha ^−1^)	95% CI for difference in mean between ESM and FD stock change (Mg C ha ^−1^)	t-statistic	Hedges’ g effect size	p-value
CMT	0–15	4.18	1.82, 6.54	3.95	2.11	0.003
	15–30	1.38	−2.95, 5.72	0.76	0.41	0.472
	30–60	0.17	−6.45, 6.80	0.06	0.03	0.954
	60–100	−0.85	−5.49, 3.80	−0.41	−0.22	0.694
	100–200	−2.99	−10.85, 4.87	−0.87	−0.46	0.409
CMT + WCC	0–15	4.07	1.58, 6.56	3.87	2.06	0.006
	15–30	2.37	−1.76, 6.49	1.32	0.70	0.223
	30–60	1.30	−2.40, 5.00	0.79	0.42	0.451
	60–100	−1.93	−8.02, 4.17	−0.71	−0.38	0.494
	100–200	−3.47	−19.67, 12.74	−0.48	−0.26	0.642
OMT	0–15	4.59	0.66, 8.52	2.95	1.57	0.030
	15–30	2.39	−0.13, 4.90	2.13	1.14	0.060
	30–60	−2.16	−5.54, 1.22	−1.44	−0.77	0.183
	60–100	−1.13	−5.91, 3.64	−0.53	−0.28	0.606
	100–200	−2.34	−16.00, 11.31	−0.39	−0.21	0.708

^a^The difference in mean between ESM and FD is calculated as ESM-FD. Under the assumption that the stock change estimated by ESM is correct (see Methods), the difference between ESM and FD can be regarded as the error from FD

^b^Treatment abbreviations are as follows: CMT is conventional maize-tomato; CMT + WCC is maize-tomato with cover crop; OMT is organic maize-tomato with cover crop

At depth, particularly at 100–200 cm, ESM estimated either a larger decrease or smaller increase in stock than FD, though these changes were not statistically significant and were associated with a large degree of variability (Table 3, Fig. 2). Figure 2 illustrates the dispersion of the underlying datapoints with estimates of SOC change on both sides of 0. Similar patterns emerge across all treatments in subsurface soils (30–200 cm): the dispersion of the data is large with wide confidence intervals for both FD and ESM estimates of change (Table 3, Fig. 2).

#### Wheat treatments: carbon concentration and bulk density change from 1993 to 2012

As with the maize treatments, the largest changes in bulk density in the wheat treatments occurred in the surface layers, particularly at 0–15 cm. There was strong evidence for decreases in bulk density at 0–15 cm for all treatments, ranging from a 10% decrease of 0.15 g cm^−3^ (*g* = −*1.82; p* = *0.014*) in IW to a 19% decrease of 0.27 g cm^−3^ (*g* = −*3.34; p* = *0.0001*) for RW + WCC (Additional file 1: Tables S3 and S7, Fig. S3). Nearly all treatments experienced bulk density decreases at 15–30 cm with moderate to strong effect sizes (Additional file 1: Table S7). In all treatments, the 30–60 cm intervals saw small increases in bulk density (up to a 6% increase in IW + N) with moderate to strong effect sizes (Additional file 1: Tables S3 and S7). Minimal changes in bulk density occurred below 60 cm with negligible effect sizes (Additional file 1: Table S7).

In contrast to the Century maize data, changes in carbon concentrations for Century wheat data were smaller in magnitude between 1993 and 2012 at 0–15 cm. Trends of positive increases in carbon concentration were observed in in IW, IW + N, RW + N, and RW + WCC, while a decrease was observed in RW (Additional file 1: Tables S3 and S8, Fig. S4). Below 15 cm, the effect sizes of carbon concentration change were also negligible to small in all treatments, with some treatments experiencing decreases and others experiencing increases (Additional file 1: Table S8, Fig. S4). Overall, the variability of change for carbon concentrations across most depths and treatments was large, as indicated by underlying observations and confidence intervals overlapping with 0 in Additional file 1: Fig. S4 (Additional file 1: Table S8). For average bulk density and carbon concentrations, see Additional file 1: Table S7. For average bulk density and carbon concentrations see Additional file 1: Table S9, and for statistical output, see Additional file 1: Tables S7 and S8.

#### Wheat treatments SOC stock change from 1993 to 2012

**Fig. 3:**
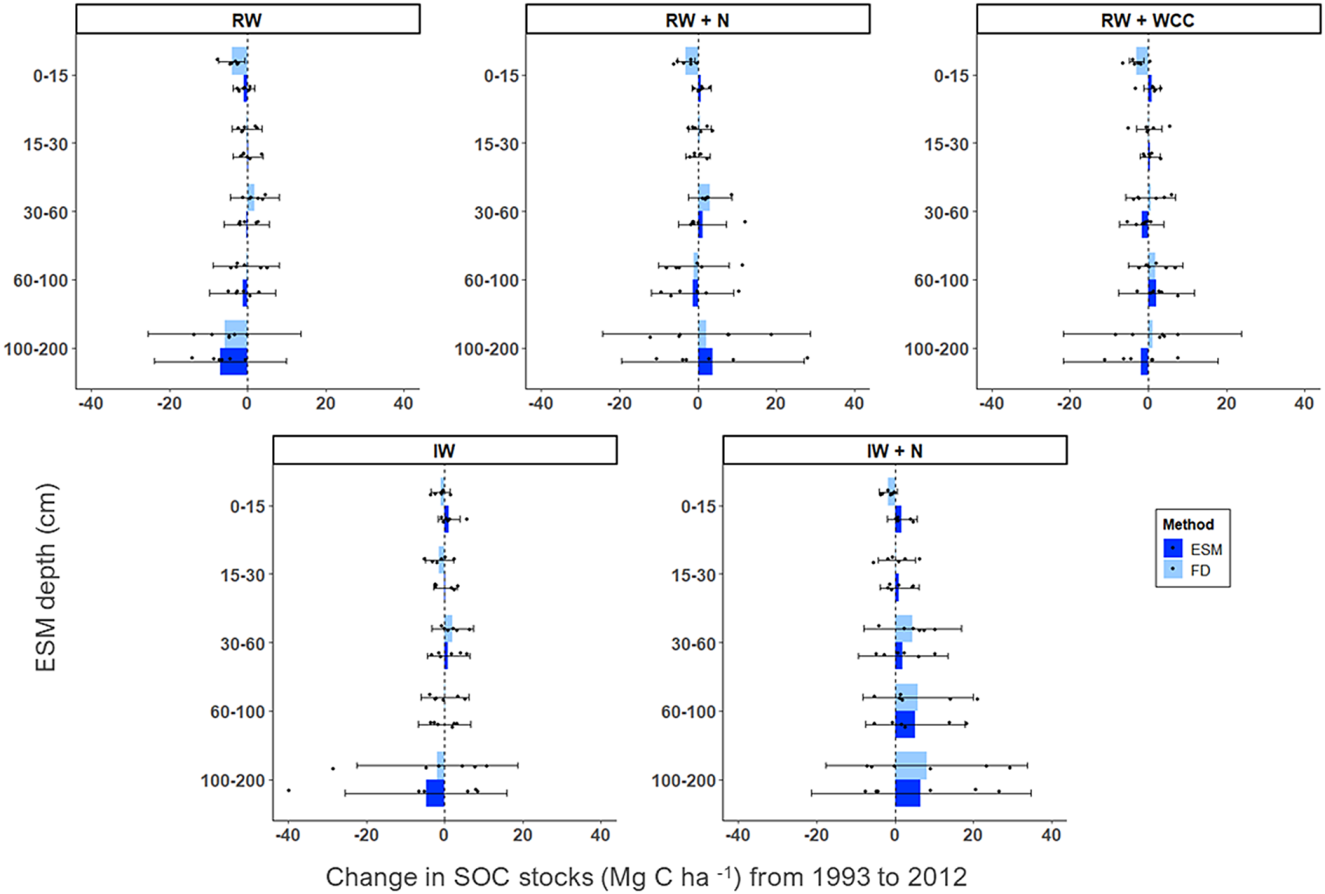
Century wheat SOC stock change from 1993 to 2012 as estimated by FD and ESM. Average change from 1993 to 2012 in SOC stocks (Mg C ha^−1^) by soil depth in each wheat Century treatment (n = 6 per treatment) as estimated by both FD and ESM. IWC is irrigated wheat–fallow with winter supplemental irrigation and no fertilizer inputs, IWF is irrigated wheat–fallow with supplemental irrigation and N fertilizer, RWC is rainfed wheat–fallow control with no additional inputs, RWF is rainfed wheat–fallow and N fertilizer, and RWL is rainfed wheat–fallow with WCC planted after wheat harvest and terminated before summer fallow. We report the results as “ESM depth” intervals [8]. These intervals are the depths represented by the reference soil masses used for the ESM calculation. The reference mass is defined as the mass from the initial fixed depth sample averaged across treatment replicates. Reference masses for each treatment and depth interval can be found in Additional file 1: Table S1a. Error bars represent 95% confidence intervals

All wheat treatments demonstrated strong evidence of differences between ESM- and FD-based estimates of SOC stock change in surface soils, with ESM consistently estimating larger stock increases and smaller decreases than FD (Table 4, Fig. 3). Notably, the direction of change for ESM and FD estimates for 0–15 cm was opposite in all treatments except for RW, where FD and ESM both estimated stock decreases (Fig. 3). For instance, in IW + N, ESM estimated a 6% increase of 1.83 (95% CI [−1.98, 5.64]) Mg C ha^−1^ and FD estimated a 5% decrease of 1.73 (95% CI [−3.99, 0.54]) Mg C ha^−1^ (Table 4 and Additional file 1: Table S3). While ESM and FD both estimated stock decreases for RW, the magnitude of difference was large (by an average of 3.14 Mg C ha^−1^; *g* = *1.71, p* = *0.011*) (Fig. 3).

**Table 4:** T-test results with confidence intervals for the difference between Century wheat ESM and FD mean stock change estimates

Treatment^b^	ESM depth (cm)	Difference in mean between ESM and FD stock change^a^ (Mg C ha ^−1^)	95% CI for difference in mean between ESM and FD stock change (Mg C ha ^−1^)	t-statistic	Hedges’ g effect size	p-value
IW	0–15	2.23	−0.45, 4.90	1.87	1.00	0.093
	15–30	1.50	−1.94, 4.94	0.97	0.52	0.353
	30–60	−1.06	−5.05, 2.93	−0.60	−0.32	0.565
	60–100	−0.12	−4.29, 4.06	−0.06	−0.03	0.950
	100–200	−2.90	−24.19, 18.40	−0.31	−0.16	0.766
IW + N	0–15	3.55	1.35, 5.76	3.65	1.95	0.005
	15–30	0.66	−3.90, 5.21	0.33	0.17	0.752
	30–60	−2.50	−9.32, 4.32	−0.82	−0.44	0.433
	60–100	−0.66	−12.84, 11.53	−0.12	−0.06	0.907
	100–200	−1.47	−20.70, 17.75	−0.17	−0.09	0.868
RW	0–15	3.14	0.91, 5.38	3.21	1.71	0.011
	15–30	0.36	−2.13, 2.85	0.32	0.17	0.755
	30–60	−2.10	−4.92, 0.72	−1.66	−0.88	0.128
	60–100	−1.03	−5.33, 3.27	−0.54	−0.29	0.602
	100–200	−1.00	−7.00, 5.00	−0.37	−0.20	0.718
RW + N	0–15	3.95	1.49, 6.40	3.68	1.96	0.006
	15–30	−0.45	−3.04, 2.13	−0.40	−0.21	0.700
	30–60	−1.96	−7.71, 3.80	−0.79	−0.42	0.452
	60–100	−0.41	−9.51, 8.68	−0.10	−0.05	0.921
	100–200	1.64	−14.49, 17.78	0.23	0.12	0.824
RW + WCC	0–15	3.93	1.05, 6.81	3.04	1.62	0.013
	15–30	0.34	−3.30, 3.97	0.22	0.12	0.833
	30–60	−2.33	−6.65, 2.00	−1.25	−0.67	0.248
	60–100	0.33	−4.08, 4.74	0.17	0.09	0.871
	100–200	−3.00	−11.06, 5.06	−0.83	−0.44	0.426

^a^The difference in mean between ESM and FD is calculated as ESM-FD. Under the assumption that the stock change estimated by ESM is correct (see Methods), the difference between ESM and FD can be regarded as the error from FD

^b^Treatment abbreviations are as follows: IW is irrigated wheat-fallow; IW + N is irrigated wheat fallow with fertilizer; RW is rainfed wheat-fallow; RW + N is rainfed wheat-fallow with fertilizer; RW + WCC is rainfed wheat with cover crops after wheat harvest and summer fallow

At 15–30 cm, the difference between ESM and FD change estimates ranged from negligible to moderate (Table 4). Differences between the two approaches were larger at 30–60 cm, with ESM, on average, estimating smaller stock changes than FD across all treatments (Fig. 3). At depths greater than 60 cm, both change in stocks as estimated by FD and ESM and the difference between those changes were variable as confidence intervals increased with depth and overlapped with 0 (Table 4, Fig. 3).

### Wisconsin Integrated Cropping Systems Trial (WICST)

#### WICST treatments: carbon concentration and bulk density change from 1989 to 2009

WICST bulk density generally increased across all treatments at 0–15 cm with changes ranging from a 4% increase of 0.05 g cm^−3^ in MAAA (95% CI [0.02, 0.08]) to a 19% increase of 0.21 g cm^−3^ in MS (95% CI [0.14, 0.28]) (Additional file 1: Tables S3 and S10, Fig. S5). At 15–30 cm, similar patterns of increased bulk density were observed across treatments with Maize increasing by 6.5% (0.03 g cm^−3^ (95% CI [−0.02, 0.08]), MS increasing by 5.5% (0.07 g cm^−3^ (95% CI [0.03, 0.11]), and MSW increasing by 4.5% (0.06 g cm^−3^ (95% CI [0.03, 0.09]) (Additional file 1: Tables S3 and S10). Bulk density was assumed to stay the same in the original work and so was not measured at 30–60 cm and 60–90 cm; thus, no change data were available for our analysis.

Carbon concentration changes in the WICST treatments also contrasted with those of the Century treatments. On average, carbon concentration decreased for nearly all treatments at nearly every depth (Additional file 1: Table S11, Fig. S6). Maize and MSW saw more pronounced losses in surface depths and smaller losses as depth increased, while MOA exhibited the opposite pattern. Like the Century wheat treatments, changes in the WICST carbon concentrations exhibited a large degree of variability (Additional file 1: Fig. S6). For average bulk density and carbon concentrations, see Additional file 1: Table S12, and for statistical output, see Additional file 1: Tables S10 and S11.

#### WICST stock change from 1989 to 2009

**Fig. 4:**
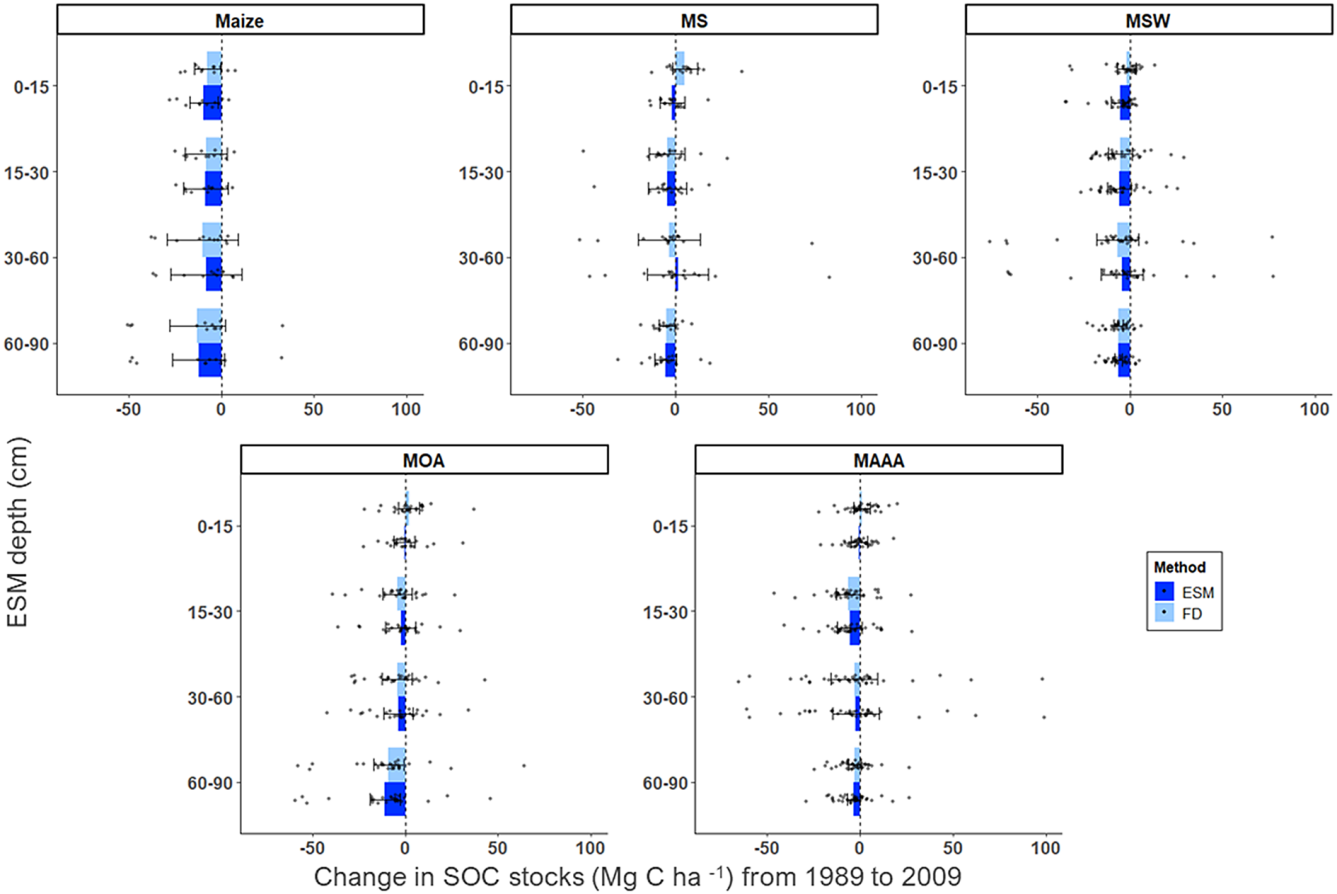
WICST SOC stock change from 1989 to 2009 as estimated by FD and ESM. Average change from 1989 to 2009 in SOC stocks (Mg C ha^−1^) by soil depth in each WICST treatment (n = 12 for Maize, n = 18 for MS, n = 33 for MSW, n = 39 for MAAA, and n = 27 for MOA) as estimated by both FD and ESM. Maize is a high-external input, continuous corn system, MS is a moderate, external input, no-till corn-soybean system, MSW is an organic corn–soybean–winter wheat with interseeded red clover system, MAAA is a high-input corn–alfalfa system, and MOA is an organic oats/alfalfa-corn system. We report the results as “ESM depth” intervals [8]. These intervals are the depths represented by the reference soil masses used for the ESM calculation. The reference mass is defined as the mass from the initial fixed depth sample averaged across treatment replicates. Reference masses for each treatment and depth interval can be found in Additional file 1: Table S1a. Error bars represent 95% confidence intervals

Both ESM and FD estimated decreases in SOC stocks for nearly all treatments and depth intervals, and the magnitude of difference between the two methods was small, except for 0–15 cm in the MS treatment (where ESM was smaller than FD by 6.98 Mg C ha^−1^ (*g* = −*0.77; p* = *0.024*) (Table 5, Fig. 4). MS’s 0–15 cm and 30–60 cm, MOA’s 0–15 cm, and MAAA’s 0–15 cm were the only depths in which the direction of change for FD and ESM estimates was opposite. However, all confidence intervals for these depths overlapped with 0 and effect sizes for MS’s 30–60 cm, MOA’s 0–15 cm, and MAAA’s 0–15 cm were small (Table 5). For the rest of the depths and treatments, FD and ESM SOC stock change estimates were similar in magnitude and direction (Table 5, Fig. 4). There was substantial dispersion among datapoints for some of the treatments, particularly MAAA, MOA, MS and MSW. For these treatments, some of the stock changes as estimated by both FD and ESM were of similar magnitude but opposite in direction (e.g., either losses or gains of ~ 50 Mg C ha^−1^) (Fig. 4).

**Table 5:** T-test results with confidence intervals for the difference between WICST ESM and FD mean stock change estimates

Treatment^b^	ESM depth (cm)	Difference in mean between ESM and FD stock change^a^ (Mg C ha ^−1^)	95% CI for difference in mean between ESM and FD stock change (Mg C ha ^−1^)	t-statistic	Hedges’ g effect size	p-value
Maize	0–15	−2.17	−10.15, 5.81	−0.56	−0.22	0.579
	15–30	−0.18	−8.26, 7.90	−0.05	−0.02	0.964
	30–60	1.79	−10.59, 14.17	0.30	0.12	0.767
	60–90	0.75	−19.90, 21.39	0.07	0.03	0.941
MS	0–15	−6.98	−12.98, -0.98	−2.37	−0.77	0.024
	15–30	0.22	−9.11, 9.55	0.05	0.02	0.962
	30–60	4.72	−12.40, 21.84	0.56	0.18	0.579
	60–90	−0.86	−6.90, 5.18	−0.29	−0.10	0.773
MSW	0–15	−3.55	−8.03, 0.94	−1.58	−0.38	0.119
	15–30	−0.33	−5.92, 5.25	−0.12	−0.03	0.905
	30–60	2.54	−10.84, 15.92	0.38	0.09	0.705
	60–90	0.09	−2.90, 3.08	0.06	0.01	0.953
MAAA	0–15	−1.51	−4.83, 1.82	−0.90	−0.20	0.370
	15–30	0.74	−4.75, 6.23	0.27	0.06	0.790
	30–60	0.70	−12.06, 13.47	0.11	0.02	0.913
	60–90	−0.48	−4.62, 3.66	−0.23	−0.05	0.818
MOA	0–15	−2.34	−7.90, 3.22	−0.84	−0.23	0.403
	15–30	1.55	−5.50, 8.60	0.44	0.12	0.661
	30–60	0.63	−7.73, 8.99	0.15	0.04	0.881
	60–90	−1.97	−14.28, 10.33	−0.32	−0.09	0.749

^a^The difference in mean between ESM and FD is calculated as ESM-FD. Under the assumption that the stock change estimated by ESM is correct (see Methods), the difference between ESM and FD can be regarded as the error from FD

^b^Treatment abbreviations are as follows: Maize is continuous maize with high external inputs; MS is moderate external input, no-till maize-soybean; MSW is organic maize-soybean-wheat followed by cover crop; MAAA is maize-alfalfa-alfalfa-alfalfa rotation; MOA is organic maize-oats/alfalfa/alfalfa rotation

#### The relationship between changes in bulk density to differences between ESM and FD

In order to understand whether there was a relationship between the change in bulk density and the difference in stocks estimated by ESM and FD in the top 30 cm of the soil, we calculated a correlation coefficient (*r)* to determine the strength of the association between the SOC stock lost or gained due to the change in bulk density and the difference between the ESM and FD stocks at t1 (2012 for the Century data and 2009 for the WICST data). We found the strongest association for the Century data, with an *r* of 0.90 for the maize treatments and an *r* of 0.88 for the wheat treatments. The association in the WICST data was not as strong, with an *r* of 0.53.

#### Crediting outcomes

To better understand the potential crediting implications of using alternative SOC accounting methods and sampling depths to estimate SOC stock change, we estimated the number of credits that would have been issued to a hypothetical 50-ha field under each management treatment in the WICST and Century experiments at 0–15, 15–30, and 30–60 cm. Overall, the Century maize treatments received more credits with ESM than with FD. CMT + WCC received credits at 0–15 cm and 15–30 cm and OMT received credits at all three depths with ESM (Table 6). In the Century wheat treatments, FD resulted in more credits overall, with credits allocated to the 30–60 cm depths; however, with ESM, all the wheat treatments, except for RW, received credits with ESM at 0–15 cm. The WICST treatments did not receive credits with ESM across all treatments, but FD resulted in credits for MS and MOA at 0–15 cm depth.

**Table 6:** Crediting outcomes of calculating SOC stocks with ESM versus FD at 0–15, 15–30, and 30–60 cm, using BCarbon’s “difference between means” methodology

Experiment	Treatment	ESM depth (cm)	ESM 50% LCL^a^ (t CO2_e_ ha^−1^)	FD 50% LCL^a^ (t CO2_e_ ha^−1^)	Number of credits for 50 ha field over 20 year crediting period using ESM	Number of credits for 50 ha field over 20 year crediting period using FD
Century Maize	CMT	0–15	−0.16	−16.59	0	0
	CMT	15–30	−2.53	−10.26	0	0
	CMT	30–60	−12.68	−15.26	0	0
	CMT + WCC	0–15	13.37	−3.07	668	0
	CMT + WCC	15–30	2.11	−8.98	105	0
	CMT + WCC	30–60	−20.34	−25.60	0	0
	OMT	0–15	33.74	13.03	1687	651
	OMT	15–30	13.56	3.70	678	185
	OMT	30–60	0.41	8.42	21	421
Century Wheat	IW	0–15	1.59	−7.09	80	0
	IW	15–30	−3.00	−9.78	0	0
	IW	30–60	−2.71	1.62	0	81
	IW + N	0–15	2.60	−9.73	130	0
	IW + N	15–30	−1.88	−4.30	0	0
	IW + N	30–60	−6.09	2.81	0	141
	RW	0–15	−7.02	−20.48	0	0
	RW	15–30	−4.16	−5.57	0	0
	RW	30–60	−8.10	−0.21	0	0
	RW + N	0–15	0.36	−15.26	18	0
	RW + N	15–30	−3.79	−1.90	0	0
	RW + N	30–60	−2.78	5.64	0	282
	RW + WCC	0–15	1.13	−14.38	56	0
	RW + WCC	15–30	−1.21	−3.53	0	0
	RW + WCC	30–60	−14.08	−5.40	0	0
WICST	Maize	0–15	−49.19	−39.73	0	0
	Maize	15–30	−50.56	−48.77	0	0
	Maize	30–60	−59.55	−66.60	0	0
	MS	0–15	−16.97	11.03	0	551
	MS	15–30	−32.17	−32.20	0	0
	MS	30–60	−17.74	−37.10	0	0
	MSW	0–15	−28.51	−14.50	0	0
	MSW	15–30	−31.26	−30.01	0	0
	MSW	30–60	−32.08	−42.12	0	0
	MAAA	0–15	−8.63	−2.57	0	0
	MAAA	15–30	−31.66	−34.30	0	0
	MAAA	30–60	−27.31	−30.00	0	0
	MOA	0–15	−9.32	0.12	0	6
	MOA	15–30	−21.53	−27.29	0	0
	MOA	30–60	−26.26	−28.80	0	0

^a^Lower Confidence Level (LCL) as determined by BCarbon’s methodology [24]

## Discussion

### FD vs. ESM

The long-term datasets used for this analysis underscore the differences in SOC stock change that arise from accounting on a FD versus ESM basis, particularly for surface soils (0–30 cm). As described in von Haden et al. (2020) [8], the primary issues with using FD to calculate SOC stocks are: (1) sampling to a fixed-depth does not account for the change in soil mass that occurs when bulk density changes over time and (2) sampling to a fixed-depth does not account for changes in soil volume when soil organic matter changes over time. As such, the ESM method can provide more reliable temporal stock comparisons than the FD approach, as long as potential error introduced by ESM is minimized with best practices (e.g., multiple depth increments and cubic spline interpolation) [8, 12, 13].

Receiving payments for credits from the soil carbon market typically requires a change in management practice. However, since management practices can impact bulk density differently, a change in practice can change the mass of soil sampled under the FD method, leading to SOC stock change errors [25–30]. For instance, following the adoption of no till, Xiao et al. [10] found consistent increases in bulk density in surface soils (0–20 cm), which led to the overestimation of SOC stocks with FD in surface layers. Similarly, in the WICST MS no-till treatment, bulk density increased by 19% in the top 15 cm, such that the FD method estimated a SOC stock increase despite a decrease in C concentration, whereas the ESM method correctly estimated a stock decrease (Table 5 and Additional file 1: Table S3). Yet the effect of different tillage systems on bulk density is variable and highly context-dependent, and bulk density may not always increase under no-till [31]. Blanco-Canqui and Ruis [32] found mixed impacts of no-till on bulk density, where bulk density increased, decreased, or did not change across different studies; this could be due to a number of environmental and temporal factors, including number of years under no-till and soil texture. Given these context specific changes in bulk density, the ESM method should be used for comparable accounting across fields.

Although bulk density changes are most often discussed in conjunction with tillage, other management practices, such as organic amendments, may also influence bulk density [30]. In the Century Experiment, the addition of compost and cover crop residues in the CMT + WCC and OMT treatments may have led to reductions in bulk density over time. Despite increases in carbon concentration, the reduction in soil mass in surface soils caused by this decrease in bulk density after 20 years resulted in SOC stock losses (or very small increases) as estimated by the FD method. Overall, the larger the bulk density decrease, the greater the difference between ESM and FD (FD error) and underestimation of stock by FD. For example, in CMT’s 0–15 cm, bulk density decreased by nearly 19% while C concentration increased by 3%. In this case, the FD percent error (assuming ESM is the correct method) was well over 100% (Table 3 and Additional file 1: Table S3). In a less extreme example, OMT had a 50% FD error rate; bulk density decreased by 16%, yet C concentration increased by 41%. While the bulk density decrease was large, the huge increase in C concentration moderated the influence of bulk density, resulting in a less extreme, though still sizeable error rate (Table 3 and Additional file 1: Table S3). Indeed, the difference between the FD and ESM estimates of stock change was strongly associated with the change in bulk density in the top 30 cm of all the maize and wheat treatments (*r* = 0.90 for maize, 0.88 for wheat).

The patterns observed in the WICST data contrasted with those in the Century data. Bulk density increased in surface soils while carbon concentration decreased across nearly all treatments and depth intervals. The difference between the experimental results from WICST and Davis highlights the differences in underlying context between the two sites related to soil texture and initial carbon concentrations. For instance, despite being classified as silt and silty-clay loams, the Century soils are coarser as evidenced by larger reference soil masses for Century (Additional file 1: Tables S1a–c) and higher sand content (21% vs. 7% for WICST) and have much lower carbon content than those at WICST. Furthermore, the input of large amounts of organic matter into the Century soils likely resulted in large bulk density decreases and increases in carbon concentrations. As a result, FD had large error and tended to underestimate the SOC stock changes. At WICST, which is located on carbon-rich Mollisols, increasing bulk density and decreasing carbon concentrations corresponded to a decrease in both ESM and FD stock estimates (Additional file 1: Table S3), with smaller differences between the two accounting approaches (Table 5). Whereas more soil may have been sampled due to bulk density increasing in in the top 30 cm for most treatments, the comparatively greater reductions in carbon concentrations effectively tempered the impact of the bulk density increase, resulting in less pronounced differences between ESM and FD accounting for the WICST soils (Table 5 and Additional file 1: Table S3). For instance, in the Maize treatment’s 0–15 cm, C concentration decreased by nearly 19% while bulk density increased by 6.5% (Additional file 1: Table S3); the resulting FD error was notably smaller (20% error rate) than the CMT and OMT example.

Our findings underscore that compared to the FD approach, ESM can result in marked differences in estimates of SOC stock change—even in direction and magnitude— yet under some soil and management contexts both approaches can yield similar estimates. The need for an ESM approach seems particularly evident in surface soils where changes in bulk density are most apparent. Despite this context dependency, we suggest that consistent use of the ESM methodology in soil carbon accounting projects will ensure comparability of stocks across time, depth, and treatments because it properly accounts for changes in bulk density. To reduce potential bias that can result from ESM calculations, we urge the use of multiple depth increments (e.g., ideally 0–15, 15–30, 30–45, 45–60 cm) when using ESM and recommend using the SimpleESM or von Haden et al.’s (2020) R script [8, 33], which use a cubic spline interpolation to account for non-linear changes in SOC and bulk density with depth [8, 12, 13].

### Sampling at-depth

Although accounting for changes in SOC stocks using an ESM versus FD approach can help reduce bias induced by changes in bulk density, there are also challenges and uncertainties associated with capturing changes in SOC stocks across the entirety of the soil profile regardless of method used. Our findings highlight the differences in SOC stocks when considering only surface soil layers (top 30 cm) vs. subsurface soils (> 30 cm). Looking at ESM stock change estimates, losses at depth appear to drive cumulative losses across the whole profile despite gains in surface soils, as highlighted by Tautges et al. [34] for the Century data [34] (Figs. 2 and 3). However, across the datasets used for our analysis, there is large spread in the underlying SOC stock data points as depth increases (Fig. 1), which is also reflected in the confidence intervals of stock change at-depth (Figs. 2, 3, 4, [5]. This large variability at depth hinders our ability to draw solid conclusions about changes occurring across the cumulative soil profile. As such, we strongly suggest following the recommendation put forth by Kravchenko and Robertson [15] to measure stocks by depth increment, rather than cumulatively, so that increased variability at certain depths does not obscure changes in less variable depths [15].

It is important to note that the processes shaping SOC dynamics at depth are different compared to surface soils and contribute to an inherent variability that makes it difficult to detect changes in SOC [15]. Heckman et al. [35] describe how weathering and transport properties, moisture and oxygen availability, reduced microbial biomass and access to organic matter, and differences in soil mineral composition all interact to influence SOC dynamics at depth. However, these dynamics remain understudied due to a lack of data on soils at depths greater than 30 cm [19].

The commonly cited challenge of detecting a small signal of change in SOC against a large standing stock (e.g., Bradford et al. [36]) is amplified for sub-surface soils: carbon concentrations are very small (although note that systems with deep roots can have larger carbon concentrations at depth), coupled with dramatic increases in soil mass. For the Century and WICST datasets, soil mass at least doubled, on average, from the surface to the deepest depths due to higher bulk density and larger depth increments (Additional file 1: Table S1a, b, and c). Therefore, even small changes in carbon concentrations in subsurface layers can seem to have an outsized influence on cumulative profile SOC stocks. In the 60–100 cm depth increment of the OMT treatment, for instance, carbon concentration change was as small as 0.04 g C kg^−1^ (95% CI [−0.85, 0.93]), which was just a 0.60% increase, after 20 years (Additional file 1: Tables S3 and S5). This small of a measured change can be extremely difficult to detect [10, 27, 37] as it is within the measurement error of commonly used SOC analytical techniques (i.e., precision ranges from 1.2 to 15.8% for loss-on-ignition, 1.6–4.2% for Walkley–Black, and 1.3–7.1% for dry combustion) [38] and begs the question of whether even state-of-the-art laboratory tools (i.e., an elemental analyzer coupled to IRMS) have sufficient precision to confidently detect changes in SOC at depth. Small rates of SOC change coupled with a short timeframe for change detection presents an additional layer of complexity, as a significant difference in a system with a slow rate of change could potentially take decades to register [38]. With most MRV protocols expecting to re-sample soils at 5-year intervals, the chance of confidently picking up real changes in SOC stocks at depth is exceedingly challenging.

Even if we assume that current tools can accurately detect small changes in carbon concentration, the challenges of accounting for SOC stock changes in subsurface soils indicate that more sampling is required. Kravchenko and Robertson [15] suggest the use of a post-hoc power analysis to identify how many samples should be or should have been taken to detect a statistically significant change in SOC. Using the CMT treatment as an example, detecting a 10% change in SOC at depth (> 30 cm) would have required at least 45 samples, albeit flaws in the post-hoc power analysis approach suggest sample numbers would likely need to be greater [39]. While taking more samples may increase the chances of detecting changes at depth, more samples will require additional effort, time and money [40], which presents logistical challenges for project developers in the voluntary carbon market.

The uncertainties and challenges associated with detecting SOC stock changes at depth mean that requiring a deeper sampling depth in soil MRV protocols may be challenging and controversial; however, current sampling requirements (e.g., only sampling down to 30 cm) may fail to capture the majority of SOC stock in agricultural soils. The data from WICST and Century coupled with results from the scientific literature suggest that sampling to 60 cm in annual row crop systems would capture the changes in bulk density that are concentrated within surface soils, any potential redistribution of SOC occurring under various management practices, and a larger proportion of the overall SOC stock [5, 10, 22, 23]. In contexts where we expect deep rooting systems to impact SOC stocks at-depth (e.g., perennial cropping and agroforestry systems), sampling even deeper may be necessary to capture the full impacts of these management interventions. Alternatively, achieving a 60 cm sampling depth may not be possible in shallow soils [19]. Yet important questions remain that demand more research to better understand how different processes and management practices influence carbon concentrations and stocks in sub-surface soils [17, 19, 35]. With carbon storage at depth gaining attention as a strategy to potentially increase stocks of more stable carbon in agricultural soils [19], this research will help to elucidate these dynamics to right-size expectations of SOC accrual across the soil profile.

### Crediting outcomes based on empirical detection of change in SOC stocks

Our credit calculation by depth reveals interesting differences in crediting outcomes between accounting methods and among different depths, as well as important questions and considerations for the soil carbon market. While credits in the carbon market would likely be calculated on a cumulative depth basis (e.g., 0–30 or 0–60 cm), we calculated credit outcomes by depth increment to show how each depth might contribute to overall credit allocation. This analysis also highlights how variability and changes in bulk density and C concentration interact to determine the number of credits received.

For example, considering only the ESM method, 4 out of the 5 Century wheat treatments received credits at 0–15 cm and no credits below 15 cm. These treatments, on average, experienced a stock increase of at least 4% at 0–15 cm (Additional file 1: Table S3). The 50% lower confidence limit (LCL) of the ESM stock differences between the two time points was positive (Table 6), meaning there was a 75% probability that the true change in stock was also positive and therefore resulted in credits. In other words, these treatments had increases in stock and less variability associated with those increases, which resulted in credits. Looking at the 15–30 cm depth increment of these treatments, we also see an average increase in ESM stock (up to 6.5% for IW + N) (Additional file 1: Table S3), yet there was more variability associated with these increases, as evidenced by the negative 50% LCL (Table 6). This is an example of how an average increase in stock with high variability resulted in no credits.

There were also clear differences between the number of credits awarded with the ESM method versus the FD method. For example, OMT received substantially more credits with ESM at 0–15 and 15–30 cm than with FD (Table 6). This difference in credits between the two methods showcases how FD underestimated the change in stock due to the substantial decreases in bulk density at these depths (Additional file 1: Tables S3 and S4). By contrast, at 30–60 cm, far more credits resulted from FD than ESM; yet when we look at the change in bulk density and C concentration (an increase in both), we can observe how FD overestimated the increase in stock due to the slight increase in bulk density. The difference in credits awarded depending on whether SOC stocks were measured using ESM or FD has implications on resulting payments to farmers.

Overall, there were many instances in which no credits were awarded regardless of method used (e.g., the WICST treatments). It is possible that the crediting outcomes could have been improved with additional sampling. Each Century treatment consisted of 6 samples, while the WICST treatments ranged from 12 to 39. We note that our crediting analysis operates at the field-scale whereas recent research highlights that the ability to detect change accurately at this scale is exceedingly challenging due to inherent variability of SOC stocks. Increased sampling density coupled with sampling across multiple fields (i.e., at a larger population scale) can deliver more accurate and robust detection of change in SOC stocks [41]. BCarbon’s protocol explains how crediting outcomes may change based on the variability in the C content of a site and number of samples taken. As such, they recommend conducting a power analysis to estimate the sample size needed to detect change in SOC or the probability of detecting change with a given number of samples [24]. For an effective power analysis, it is necessary to understand the variability in SOC within a field, which may not be known if the site has not been sampled before. Ultimately, they note that “the user will need to assess whether the additional credit warrants the cost of the additional sampling.” While this emphasizes the importance of taking enough samples to detect change in SOC, the price of carbon should reflect the work required to accurately estimate the change.

Regardless of the number of samples taken, the crediting outcomes for both experiments beg the question of what happens to fields that show SOC stock losses at the end of a crediting period; it is currently unclear what declining SOC stocks may mean in terms of payments for participating farmers, particularly in protocols that employ a static baseline. Carbon crediting projects that only rely on empirical soil sampling to detect SOC stock changes using a static baseline do not allow one to observe instances in which eligible practices have slowed the loss of SOC compared to what would have happened under the counterfactual or “business-as-usual” (BAU) approach [42]. As a result, crediting projects with a static baseline rest on the assumption that SOC stocks will increase under eligible practices. Our data illustrate the importance of comparing an improved practiced to BAU: for example, even though SOC stocks decreased across all WICST treatments, the conventional treatment (Maize) experienced more pronounced declines than the alternative management treatments (Fig. 4). Having a business-as-usual reference could enable farmers to receive credits for slowing losses of SOC stocks under newly adopted practices. Creating adequate safeguards for farmers is critical, given that many may remain wary of participating in the market until there are more structures in place to protect their interests [43].

## Conclusion

Creating MRV protocols that deliver high integrity soil carbon credits requires SOC accounting and sampling methods that accurately reflect changes in C stocks. Although soil carbon sequestration is just one small tool in a large toolbox of climate solutions, it is important to ensure that we get it right given the urgency of climate change and the speed at which the voluntary carbon market is expanding and evolving.

We have shown that using FD, presently the most used SOC accounting method in MRV protocols, can lead to substantial errors in SOC stock change estimates due to changes in soil bulk density that occur over time and often accompany management practice shifts [4, 5, 10]. Resulting stock change differences between FD and ESM approaches, when translated into carbon credits, could determine whether or not farmers are eligible for carbon farming incentives. We also further highlight the challenges and uncertainty associated with detecting changes in SOC at depth. The lack of data for subsurface soils demands research efforts geared towards increasing our understanding of the variability of SOC stocks at depth and how management influences subsurface SOC dynamics. This will help design effective soil sampling strategies and determine the analytical precision necessary to detect SOC changes across the soil profile. A standardized approach to carbon crediting programs in annual, row crop agroecosystems would be to require ESM sampling to a depth of 60 cm with multiple depth increments (e.g., ideally 0–15, 15–30, 30–45, 45–60) to effectively capture a larger portion of the SOC stock, thereby building confidence that changes in management are not merely redistributing soil carbon and that management influences on bulk density are accurately accounted [19].

Empirically detecting change in soil carbon sequestration should be an essential requirement for soil carbon sequestration MRV. The methods employed to do so must reflect our best understanding of how to capture accurate, unbiased estimates of SOC stock change. Our analysis adds to the growing call for incorporating ESM and sampling at depths beyond 30 cm as best practice in MRV protocols [4, 8, 44].

## Methods

### Data sources

Two different datasets were used for this analysis. The first dataset comes from University of California, Davis’ Century Experiment at the Russel Ranch Sustainable Agriculture Facility (38°32′24″N, 121°52′12″W) [34]. The second dataset comes from UW Madison’s Wisconsin Integrated Cropping Systems Trial (WICST) at the UW Madison Agricultural Research Station in Arlington, WI (43°18″N, 89°20″W) [45]. These datasets were chosen because they contain long-term carbon (C) concentration and bulk density measurements, sample at depths greater than 30 cm, and represent two different U.S. geographies with different cropping systems (see below). Data were previously published in Sanford et al. [45] and Tautges et al. [34] to explore management impacts on soil carbon accrual over time [34, 45].

#### Century experiment

The Century Experiment was established as a long-term experiment in 1993 to study various aspects of wheat- and maize-based cash crop rotations common to northern California. The site is in California’s northern Central Valley and has two different types of soils (a) Yolo silt loam (Fine‐silty, mixed, superactive, nonacid, thermic Mollic Xerofluvents) and (b) Rincon silty clay loam (fine, smectitic, thermic Mollic Haploxeralfs). Average texture across all depths in the Maize treatment plots (the only data we were able to obtain) is ~ 21% sand, ~ 60% silt, ~ 19% clay for Yolo Silt Loam and ~ 22% sand, ~ 59% silt, and ~ 20% clay for the Rincon silty clay loam.

The experiment was set up as a randomized complete block design with three blocks, where two blocks were placed on the Rincon silty clay loam, and the third block on the Yolo silt loam. There were nine cropping systems in two-year rotations on 0.4 ha (64 × 64 m) replicate plots. Each cropping system was replicated six times (two plots per block) so that both crops in the two-year rotations were present within a block every year.

The maize-based systems consisted of the following treatments: (1) conventional maize–tomato with synthetic fertilizer, pesticides, and winter fallow (CMT); (2) a maize-tomato system with synthetic fertilizer, pesticide, and a winter cover crop (WCC) (CMT + WCC); and (3) certified organic maize–tomato with composted poultry manure and WCC (OMT) (see Table 1). The five wheat-based systems were (1) a rainfed wheat–fallow control with no additional inputs (RW), (2) rainfed wheat–fallow + N fertilizer (RW + N), (3) rainfed wheat–fallow with WCC planted after wheat harvest and terminated before summer fallow (RW + WCC), (4) irrigated wheat–fallow with winter supplemental irrigation and no fertilizer inputs (IW), and (5) irrigated wheat–fallow with supplemental irrigation and N fertilizer (IW + N). For further details on experimental design and treatments, see Tautges et al. [34].

#### Century soil sampling and soil carbon and bulk density determination

Soils were sampled as 3-cm inner diameter soil cores from the six replicates in all nine cropping systems. In September 1993, the samples were composited from 10 random locations within plots in depth increments of 0–15, 15–30, 30–60, 60–100, and 100–200 cm. In September 2012, 3-cm diameter cores were also collected from all six replicates of the nine cropping systems, but samples were composited from 6 random locations per plot. All sampling occurred prior to tillage.

In 1993, bulk density was collected in 0–25, 25–50, 50–100, and 100–200 cm depth increments with an 8.25-cm diameter probe. In 2012, bulk density was collected in 0–15, 15–30, 30–60, 60–100, and 100–200 cm depths, with a 4.7-cm diameter probe. For both years, cores were collected from 4 random locations within each plot. Bulk density depths from 1993 were adjusted to 2012 depths by calculating weighted averages using the two adjacent 1993 to 2012 depths.

Subsamples from well-homogenized archived soils from 1993 and 2012 were collected in 2015 for total carbon determination, which was determined by dry combustion (ECS 4010 Costech Elemental Analyzer). See Tautges et al. (2019) for further details on soil sampling, bulk density determination and lab analysis [34].

#### Wisconsin Integrated Cropping Systems Trial (WICST)

WICST is a long-term experiment that began in 1990 and consists of six cropping system systems typical of North Central USA. The site is located on Plano silt loam (fine-silty, mixed, superactive, Mesic Typic Argiudolls; 7% sand, 72% silt, 21% clay in the top 15 cm), which are deep, well-drained soils that were formed under prairie vegetation in loess deposits. The site was converted from prairie vegetation to cropland in the mid-nineteenth century.

In 1989, corn was planted across the 24 ha WICST study area to allow for baseline measurements and to improve the uniformity of crop history. The experiment is a four-block randomized complete block design with one replication of the 14 total crop phases in the six cropping systems placed in each block. Beginning in 1990, cropping system establishment was staggered to ensure that each phase of each rotation was replicated in time and space. Once the stagger was complete in 1992, every phase was present every year for all the crop rotations.

The six cropping systems at WICST include three cash-grain and three dairy-forage rotations. The grain systems include (Table 2): (1) high-external input, continuous maize (Maize); (2) moderate external input, no-till maize-soybean (MS); and (3) organic maize–soybean–winter wheat followed by cover crop (MSW). The forage systems include (1) moderate external input, maize–3 yr alfalfa (MAAA); (2) organic maize-oats/alfalfa-alfalfa (MOA); and (3) rotationally grazed pasture seeded to a mixture of red clover, timothy, smooth bromegrass, and orchardgrass (Pasture, not included in the present analysis). See Sanford et al. [45] for further details on experimental design [45].

#### WICST soil sampling and soil carbon and bulk density determination

Soils were sampled in 1989 on a 27 m × 27 m grid overlaying the 24-ha study area prior to layout of the contemporary WICST plots. Plots were 0.3 ha. Soil cores were taken using a 3.2-cm diameter probe for the 0–15 and 15–30 cm depths, and a 1.9-cm diameter probe for the 30–60, and 60–90 cm. All sampling occurred prior to tillage (Additional file 1: Table S2). At each of the sampling points, four cores were taken and homogenized by depth. In 2009, cores were taken with a 3.2-cm diameter soil probe in all plots and then divided into the same depth increments as 1989. To match the 2009 data set, the 1989 grid SOC values were converted to plot-level data by overlaying the 1990 plot map on the 1989 grid map.

In 1989, soil cores for bulk density were collected at 0–15 and 15–30 cm on the baseline grid. Bulk density for the plot level data for 1989 was obtained in the same way as described above for the SOC estimates. In 2007 and 2008, bulk density was estimated at depth increments of 0–15, 15–30, 30–60, and 60–90 cm. Since the 2007 and 2008 samples were so similar to each other, the two sampling times were averaged together to provide a robust estimate for 2009. The authors assumed that below 30 cm, there would likely be no significant or detectable change in bulk density after 20 years, therefore the same values for 30–60 cm and 60–90 cm were used for 1989 and 2009. Subsequent retroactive analysis has supported this assumption (*data not shown*).

Visible plant material was picked out of all samples and total carbon (TOC) was determined by dry combustion using a Flash EA 1112 CN Automatic Elemental Analyzer. See Sanford et al. [46] for further details on soil sampling and bulk density determination. TOC is used interchangeably with SOC at WICST as several previous assessments have revealed negligible quantities of inorganic carbon in these loess soils (< 0.05 g kg^−1^) [46].

### SOC stock and stock change calculations

We calculated SOC stocks using SimpleESM, an R function developed to calculate SOC and nitrogen stocks using three different SOC stock accounting methods: the traditional fixed-depth (FD) method, the classical equivalent soil mass (ESM) method [47, 48], and an alternative ESM method based on a “material coordinate system” [49] or “cumulative coordinates approach” [11] (see Ferchoud & Chlebowski) [33].

The fixed-depth method multiplies carbon concentration, bulk density, and the thickness, or depth (cm), of a soil layer to estimate the SOC stock. Importantly, this depth is the same at initial and subsequent sampling times. ESM methods use the soil mass of a reference soil layer to calculate the stock. The reference mass is defined as the mass from the initial fixed depth sample averaged across treatment replicates (see Additional file 1: Table S1a, b, c for ESM reference mass specific to each treatment and depth). This ensures that stock calculations are not affected by changes in bulk density, which can affect the mass of the soil sampled in a given soil layer (see von Haden et al. [8] for illustrative examples). The alternative ESM method uses a cubic spline interpolation to determine the relationship between cumulative soil mass and cumulative SOC stocks. Interpolation enables the determination of SOC stocks at a given reference mass that remains consistent over time and so avoids the bias of fixed depth approaches [8, 33].

The SimpleESM function requires the user to input carbon concentration (g kg^−1^) and bulk density (g cm^−3^) data to determine SOC stock by depth (Mg C ha^−1^), cumulative SOC stock (Mg C ha^−1^), soil mass (Mg ha^−1^), cumulative soil mass (Mg ha^−1^), and carbon concentration (g kg^−1^) on a fixed depth and ESM basis. We used an initial sampled mass (averaged by depth for each treatment) for both datasets (1993 for Century and 1989 for WICST) as the reference soil masses.

For this analysis, carbon concentration, bulk density and SOC stock changes were calculated by subtracting the t0 values from the t1 values for both experiments. Fixed-depth stock SOC estimates were compared to the alternative ESM stock estimates (for our analysis, the difference between the classical and alternative ESM approaches was negligible). Following Rovira et al. (2022), we assume that ESM is the correct method and regard the difference between ESM and FD (ESM–FD) as the error resulting from FD [12].We assume that our use of ESM is correct because we have multiple depth intervals and employ cubic spline interpolation rather than linear interpolation [8, 13]. See Fowler et al. [13] and von Haden et al. [8] for further recommendations on proper use of the ESM method to avoid any potential bias.

### Statistical analysis

As the intent of our analysis was to determine differences between ESM and FD accounting, we focused on analyzing those differences as opposed to management impacts on carbon sequestration. The original publications for the Century Experiment [34] and WICST [45] contain details and results specific to treatment impacts on SOC accrual.

For our analysis, we used the “R” package *rstatix* [50, 51] to calculate 95% confidence intervals (CI) and to perform t-tests to assess differences between time 0 (t0) and time 1 (t1) mean carbon concentrations and bulk density, as well as the difference between the mean stock changes estimated by ESM and FD accounting methods. We also calculated Hedges’ g to determine the effect size (hereinafter referred to as “*g*”), which calculates the difference in means between t0 and t1 as the number of standard deviations that separates those means. Differences were assessed by treatment and depth.

### Association between difference in FD and ESM stocks and bulk density changes

We also assessed whether there was an association between the change in bulk density from t0 to t1 and the difference in stocks estimated by ESM and FD at t1 in the top 30 cm of the soil. We focused on surface soils because that is where the bulk density changes were the greatest. First, we found the difference in bulk density from t0 to t1 and how much soil was lost or gained over time given this difference. We then calculated the SOC stock (Mg C ha^−1^) in the soil that was lost or gained, based on the t1 C concentration. The resulting stock lost or gained was compared to the difference between t1 FD and ESM stock estimates. We calculated the correlation coefficient (*r)* to determine the strength of the association between these two variables (the stock lost or gained due to the change in bulk density and difference between the ESM and FD stocks).

### Estimation of carbon credits

To demonstrate implications of carbon accounting methodology and sampling depth on crediting outcomes, we calculated the number of credits that would be issued over the 20-year sampling period for the Century and WICST experiments at 0–15, 15–30, and 30–60 cm using ESM and FD accounting methods. We chose to calculate credits by depth increment to highlight how crediting outcomes may differ depending on the amount of variability associated with changes at each depth interval. We recognize that this is not how current protocols operate and that accounting is largely based on soil samples taken at 0–30 cm depth. Although some MRV protocols generating credits for agricultural carbon sequestration require the use of both modeling (e.g., process-based models like DNDC or DayCent) and empirical sampling, the BCarbon and Australia’s Carbon Credits Methodology Determination allow credits to be calculated based on empirical sampling alone [14, 24, 52]. These protocols require measurement of soil carbon stocks at the field scale to establish a static baseline against which changes in carbon stocks are measured over time. We used the guidance presented in the BCarbon protocol to calculate SOC stock change and the number of carbon credits under their empirical sampling framework [24].

BCarbon’s protocol allows for two methods to calculate the difference in SOC between time points: the difference between means in SOC stock, which requires calculating the difference between means with a 50% CI or the mean difference using a 90% CI. We used the difference between means approach as the protocol indicates this is better suited for soils with more variable SOC content. We calculated the difference between means using the t-test function of the “R” package *rstatix* [50, 51]. The number of credits issued is then based off the 50% lower confidence interval (LCL) on the difference between means; as such, we determined the 50% CI to find the LCL following BCarbon’s methodology [24]. Using the 50% LCL as opposed to the mean difference is a more conservative approach, since it aims to prevent over-crediting 75 percent of the time [24]. Conversely, 25 percent of the time the true change is below the LCL, which could result in over-crediting. See BCarbon protocol for additional details on estimating net change in SOC stock [24].

As per the protocol, we then converted the 50% LCL from Mg C ha^−1^ to t CO_2_e ha^−1^. Although credits are typically issued on a per acre basis, we kept the units in hectares to keep units consistent with the rest of our analyses and to simulate the total amount of credits a 50-ha field would receive over the course of a 20-year crediting period. Note that BCarbon’s protocol specifies that, should average bulk density differ between the beginning and ending sampling points by more than 5 percent, additional steps need to be taken to ensure an equivalent mass of soil is compared. For illustrative purposes, we estimated credits usings stocks calculated with both ESM and FD.

### Supplementary Information

**Additional file 1: Figure S1.** Century maize bulk density change from 1993 to 2012. **Figure S2.** Century maize carbon concentration change from 1993 to 2012. **Figure S3.** Century wheat bulk density change from 1993 to 2012. **Figure S4.** Century wheat carbon concentration change from 1993 to 2012. **Figure S5.** WICST bulk density change from 1989 to 2009. **Figure S6.** WICST carbon concentration change from 1989 to 2009. **Table S1a.** Century maize ESM reference masses. **Table S1b.** Century wheat ESM reference masses. **Table S1c.** WICST ESM reference masses. **Table S2.** Typical (long-term) tillage practices at the Wisconsin Integrated Cropping Systems Trial. Practices may vary slightly in any given year depending on soil conditions and weed pressure. **Table S3.** C concentration, bulk density, ESM stock and FD stock percent change from t0 to t1 for all treatments and depths. **Table S4.** T-test results with confidence intervals for the difference in mean between 1993 and 2012 Century maize bulk density measurements. **Table S5.** T-test results with confidence intervals for the difference between 1993 and 2012 Century maize carbon concentrations. **Table S6.** Mean and standard deviation of Century maize carbon concentrations and bulk density by treatment and depth. **Table S7.** T-test results with confidence intervals for the difference between 1993 and 2012 Century wheat bulk density measurements. **Table S8.** T-test results with confidence intervals for the difference between 1993 and 2012 Century wheat carbon concentrations. **Table S9.** Mean and standard deviation of Century wheat carbon concentrations and bulk density by treatment and depth. **Table S10.** T-test results with confidence intervals for the difference between 1989 and 2009 WICST bulk density measurements. **Table S11.** T-test results with confidence intervals for the difference between 1989 and 2009 WICST carbon concentrations. **Table S12.** Mean and standard deviation of WICST carbon concentrations and bulk density by treatment and depth. **Table S13.** T-test results with confidence intervals for the difference between 1993 and 2012 Century maize ESM stock estimates. **Table S14.** T-test results with confidence intervals for the difference between 1993 and 2012 Century wheat ESM stock estimates. **Table S15.** T-test results with confidence intervals for the difference between 1989 and 2009 WICST ESM stock estimates.

## Data Availability

Data analyzed for this study will be deposited in the Dryad Digital Repository.

## Abbreviations

C: Carbon
CI: Confidence interval
CO_2_e: Carbon dioxide equivalents
ESM: Equivalent soil mass
FD: Fixed-depth
g: Hedge’s g, a standardized measure of effect size
LCL: Lower confidence level
SOC: Soil organic carbon
CMT: Conventional maize–tomato with synthetic fertilizer, pesticides, and winter fallow treatment
CMT + WCC: A hybrid system with synthetic fertilizer, pesticide, and a winter cover crop (WCC) treatment
OMT: Certified organic maize–tomato with composted poultry manure and WCC treatment
RW: Rainfed wheat–fallow control with no additional inputs treatment
RW + N: Rainfed wheat–fallow and N fertilizer treatment
RW + WCC: Rainfed wheat–fallow with WCC planted after wheat harvest and terminated before summer fallow treatment
IW: Irrigated wheat–fallow with winter supplemental irrigation and no fertilizer inputs treatment
IW + N: Irrigated wheat–fallow with supplemental irrigation and N fertilizer treatment
Maize: High-external input, continuous maize system treatment
MS: Moderate external input, no-till maize-soybean system treatment
MSW: Organic maize-soybean-wheat followed by cover crop treatment
MAAA: Maize-alfalfa-alfalfa-alfalfa rotation treatment
MOA: Organic maize-oats/alfalfa/alfalfa rotation treatment
MRV: Measurement, reporting, and verification protocols
