# Supplementary material for: The importance of accounting method and sampling depth to estimate changes in soil carbon stocks
Source: Carbon Balance Manag. 2024 Jan 26;19:2. doi: 10.1186/s13021-024-00249-1 (PMC10811869; doi:10.1186/s13021-024-00249-1)
Supplement: Supplementary file 1 — Additional file 1: Figure S1. Century maize bulk density change from 1993 to 2012. Figure S2. Century maize carbon concentration change from 1993 to 2012. Figure S3. Century wheat bulk density change from 1993 to 2012. Figure S4. Century wheat carbon concentration change from 1993 to 2012. Figure S5. WICST bulk density change from 1989 to 2009. Figure S6. WICST carbon concentration change from 1989 to 2009. Table S1a. Century maize ESM reference masses. Table S1b. Century wheat ESM reference masses. Table S1c. WICST ESM reference masses. Table S2. Typical (long-term) tillage practices at the Wisconsin Integrated Cropping Systems Trial. Practices may vary slightly in any given year depending on soil conditions and weed pressure. Table S3. C concentration, bulk density, ESM stock and FD stock percent change from t0 to t1 for all treatments and depths. Table S4. T-test results with confidence intervals for the difference in mean between 1993 and 2012 Century maize bulk density measurements. Table S5. T-test results with confidence intervals for the difference between 1993 and 2012 Century maize carbon concentrations. Table S6. Mean and standard deviation of Century maize carbon concentrations and bulk density by treatment and depth. Table S7. T-test results with confidence intervals for the difference between 1993 and 2012 Century wheat bulk density measurements. Table S8. T-test results with confidence intervals for the difference between 1993 and 2012 Century wheat carbon concentrations. Table S9. Mean and standard deviation of Century wheat carbon concentrations and bulk density by treatment and depth. Table S10. T-test results with confidence intervals for the difference between 1989 and 2009 WICST bulk density measurements. Table S11. T-test results with confidence intervals for the difference between 1989 and 2009 WICST carbon concentrations. Table S12. Mean and standard deviation of WICST carbon concentrations and bulk density by treatment and depth. Table S13. [file 13021_2024_249_MOESM1_ESM.docx]

**Supplementary figures**


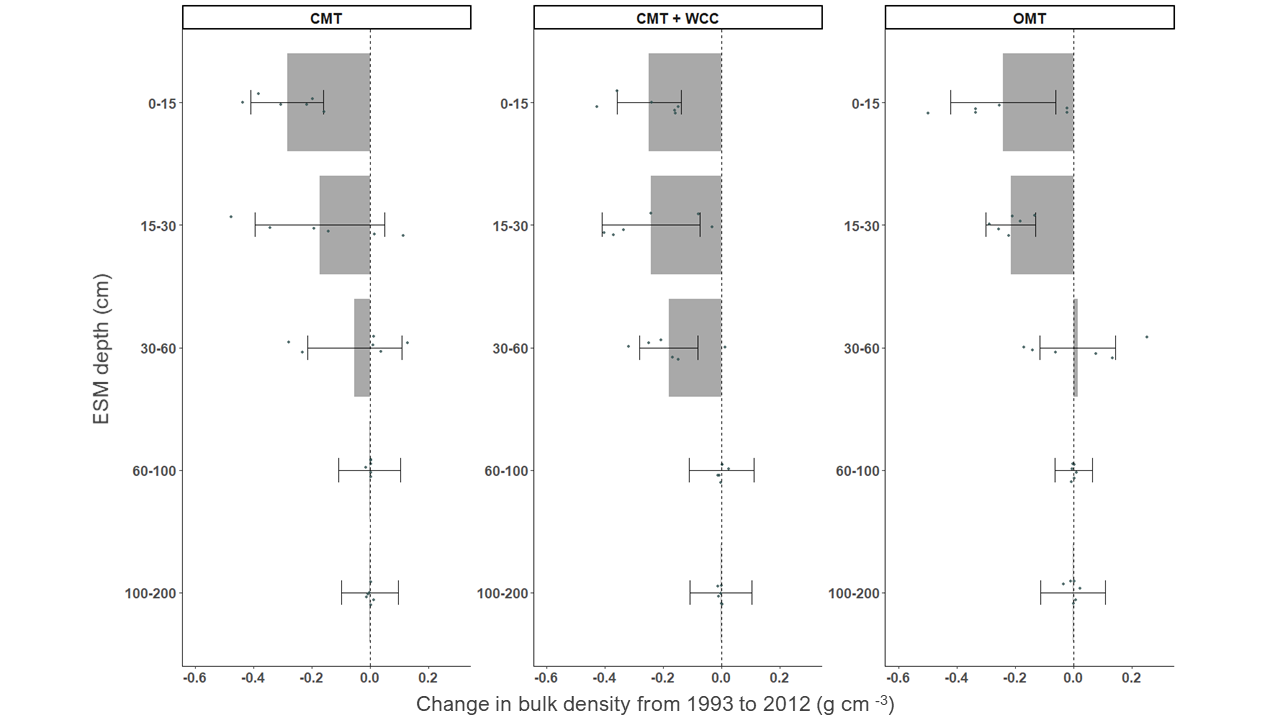
**Figure S1.** Century maize bulk density change from 1993 to 2012

**Figure S2.** Century maize carbon concentration change from 1993 to 2012


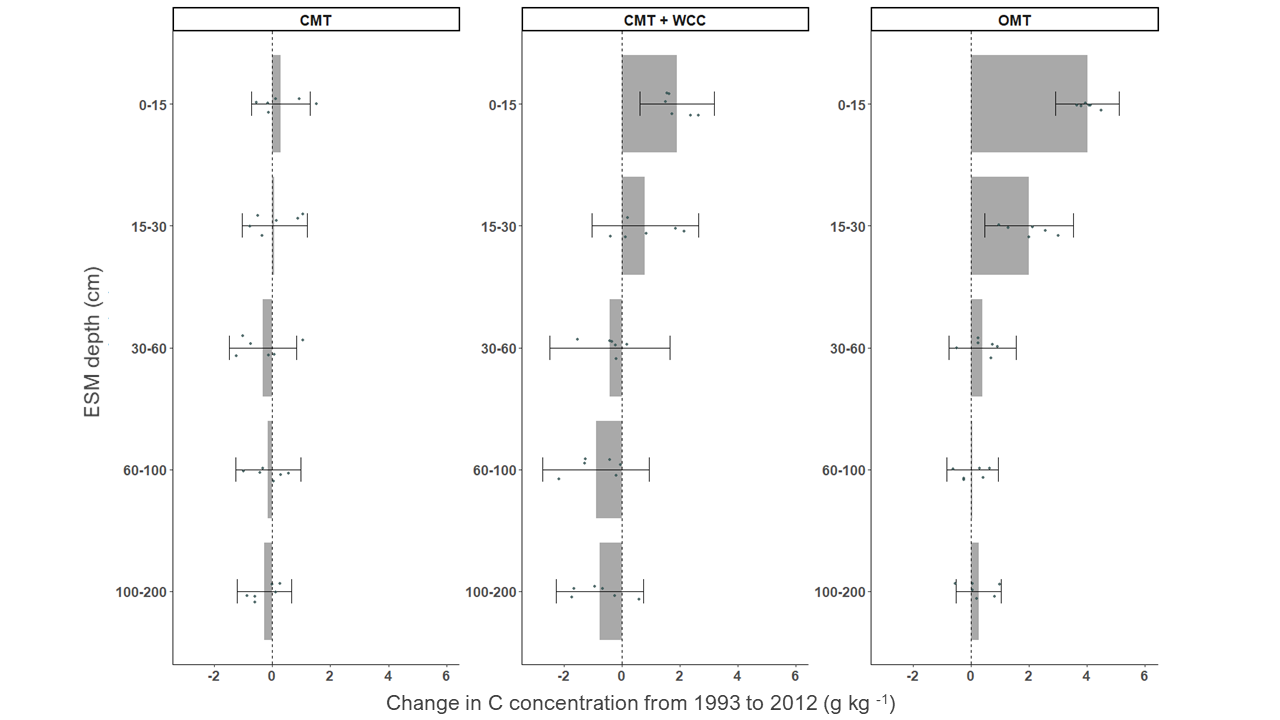


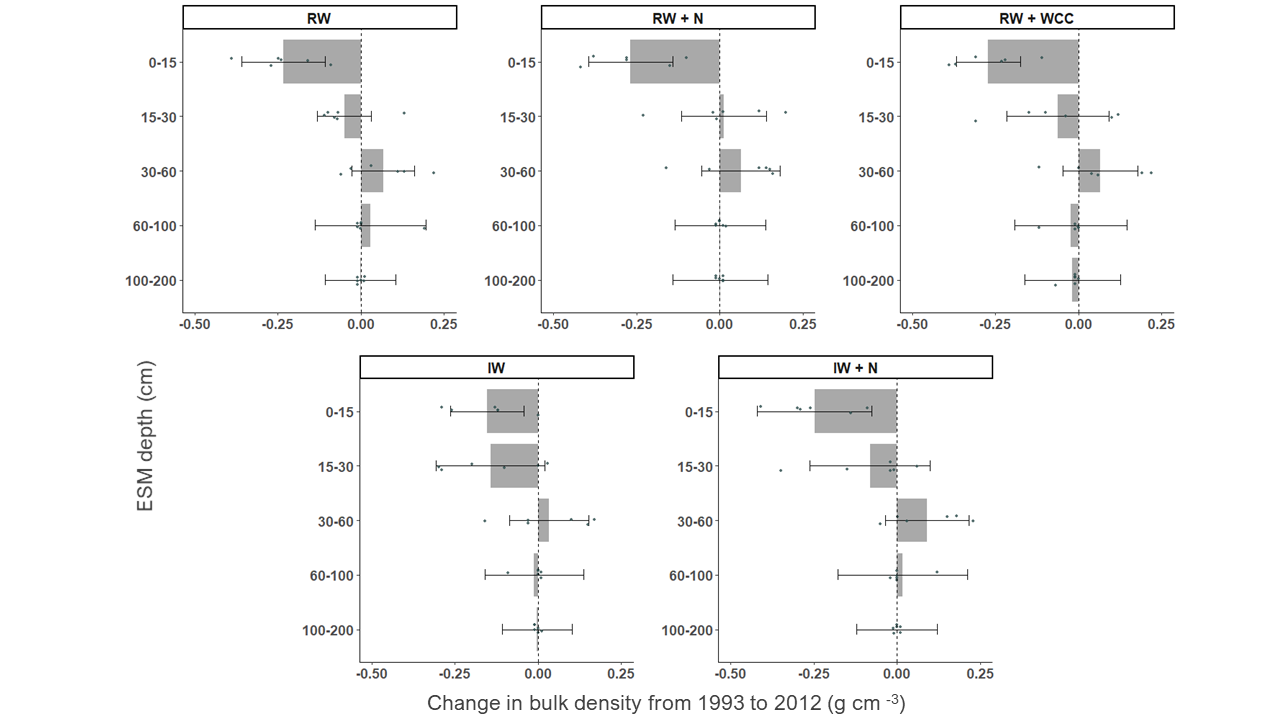
**Figure S3.** Century wheat bulk density change from 1993 to 2012

**Figure S4.** Century wheat carbon concentration change from 1993 to 2012


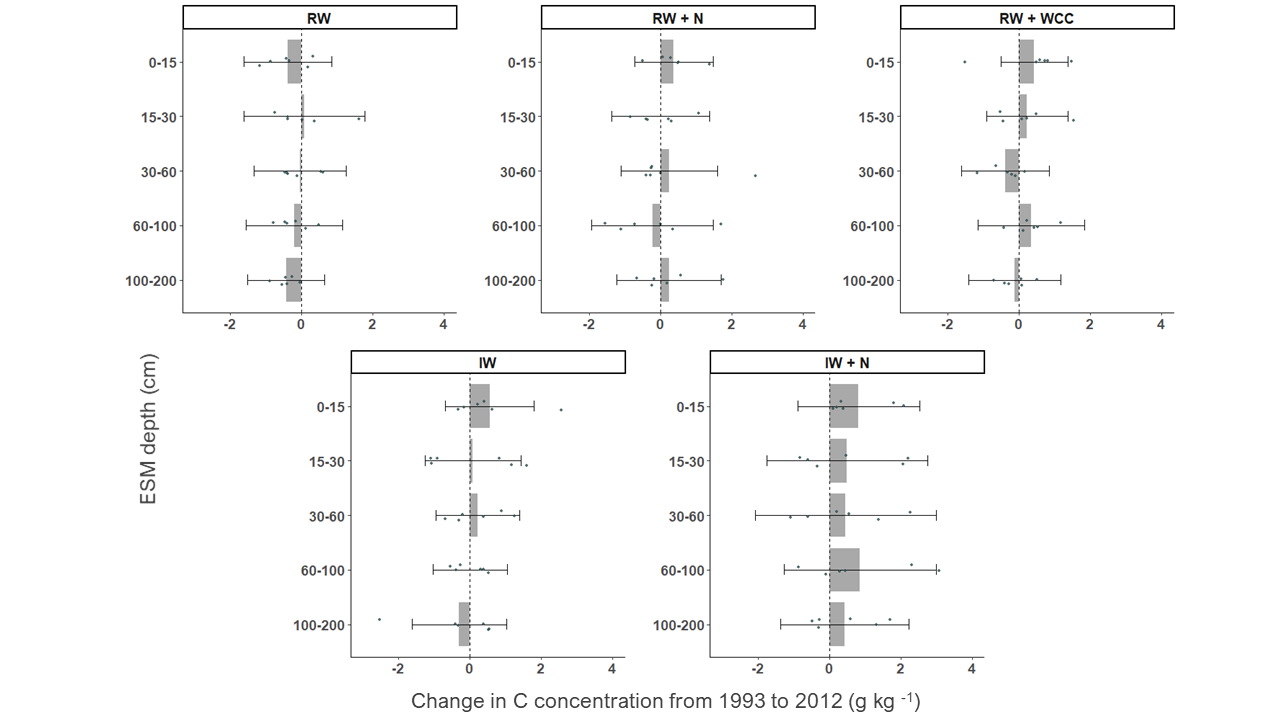


**
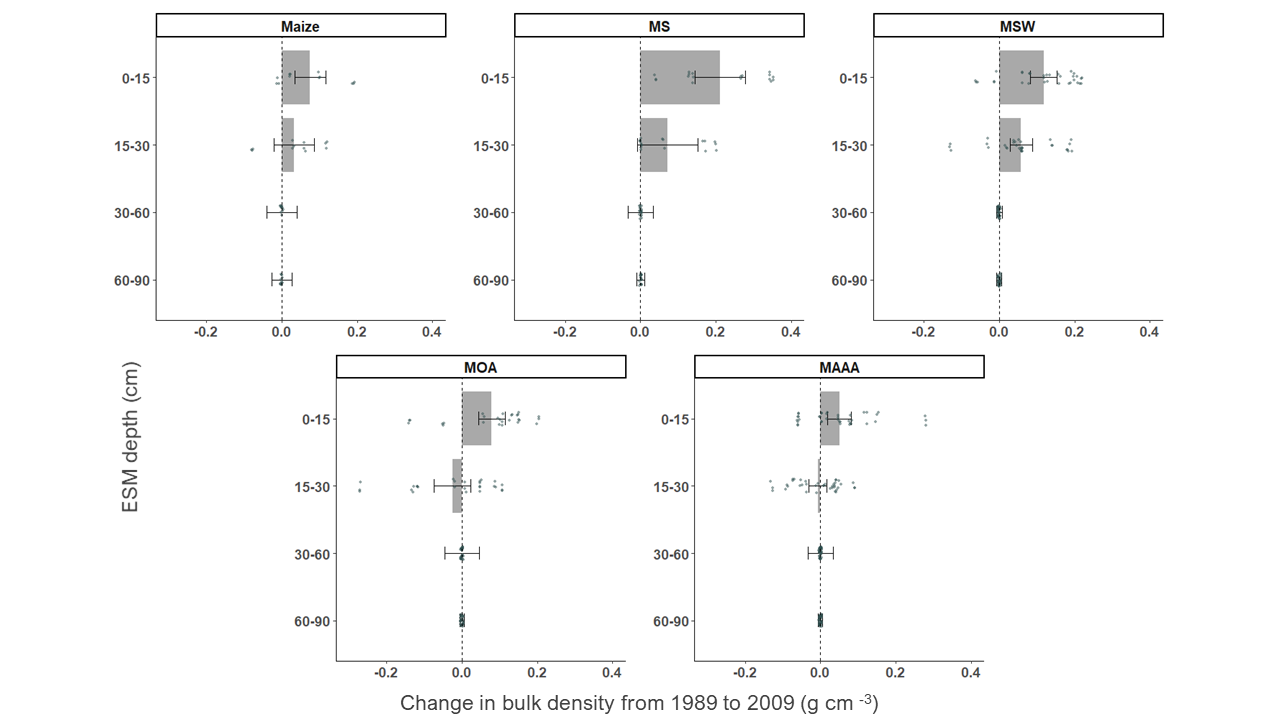
Figure S5.** WICST bulk density change from 1989 to 2009

**Figure S6.** WICST carbon concentration change from 1989 to 2009


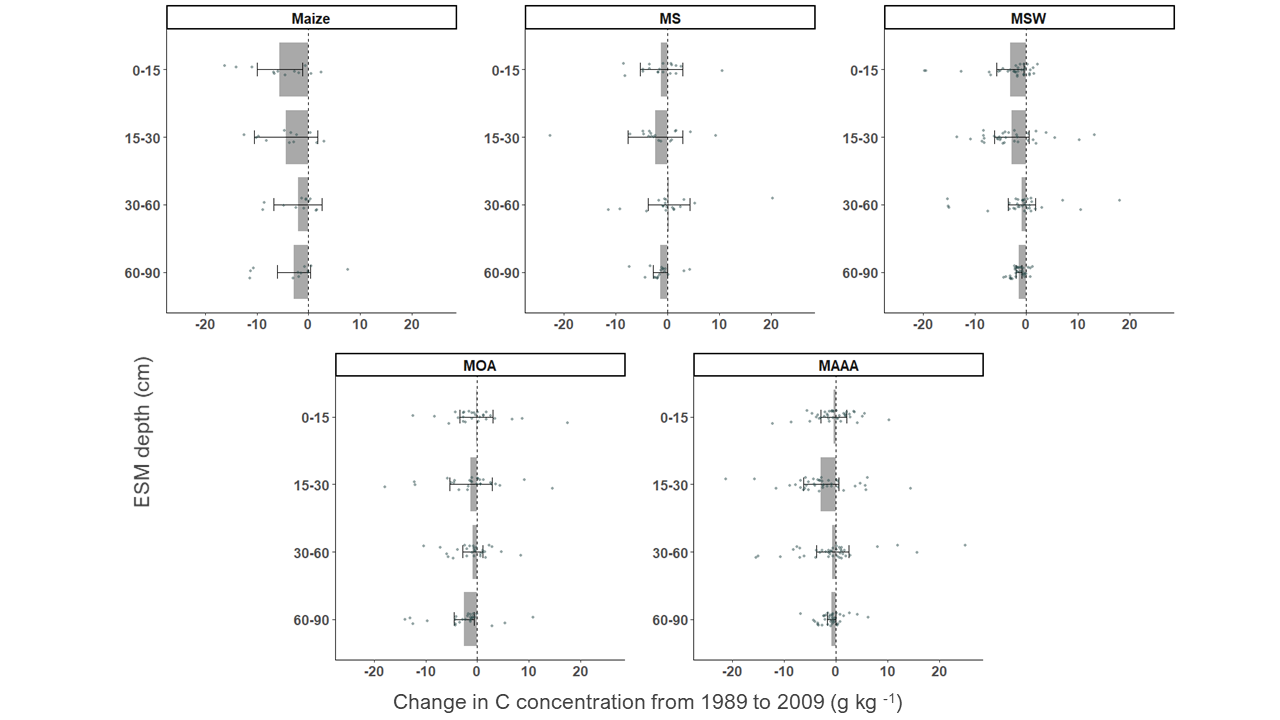


**Supplementary tables**

**Table S1a.** Century maize ESM reference masses

| **Treatment** | **Reference layer (cm)^1^** | **ESM reference mass (Mg ha ^-1^)** |
| --- | --- | --- |
| **CMT** | 0-15 | 2263 |
|  | 15-30 | 2238 |
|  | 30-60 | 4575 |
|  | 60-100 | 6340 |
|  | 100-200 | 15200 |
| **CMT + WCC** | 0-15 | 2193 |
|  | 15-30 | 2220 |
|  | 30-60 | 4710 |
|  | 60-100 | 6460 |
|  | 100-200 | 15750 |
| **OMT** | 0-15 | 2283 |
|  | 15-30 | 2225 |
|  | 30-60 | 4480 |
|  | 60-100 | 6553 |
|  | 100-200 | 16067 |

**^1^** Initial fixed depth increment

**Table S1b.** Century wheat ESM reference masses

| **Treatment** | **Reference layer (cm)^1^** | **ESM reference mass (Mg ha ^-1^)** |
| --- | --- | --- |
| **IW** | 0-15 | 2205 |
|  | 15-30 | 2205 |
|  | 30-60 | 4620 |
|  | 60-100 | 6413 |
|  | 100-200 | 15683 |
| **IW + N** | 0-15 | 2233 |
|  | 15-30 | 2198 |
|  | 30-60 | 4475 |
|  | 60-100 | 5993 |
|  | 100-200 | 15633 |
| **RW** | 0-15 | 2258 |
|  | 15-30 | 2235 |
|  | 30-60 | 4510 |
|  | 60-100 | 6127 |
|  | 100-200 | 15567 |
| **RW + N** | 0-15 | 2218 |
|  | 15-30 | 2235 |
|  | 30-60 | 4520 |
|  | 60-100 | 6120 |
|  | 100-200 | 15850 |
| **RW + WCC** | 0-15 | 2180 |
|  | 15-30 | 2158 |
|  | 30-60 | 4515 |
|  | 60-100 | 6467 |
|  | 100-200 | 15300 |

**^1^** Initial fixed depth increment

**Table S1c.** WICST ESM reference masses

| **Treatment** | **Reference layer (cm)^1^** | **ESM reference mass (Mg ha ^-1^)** |
| --- | --- | --- |
| **Maize** | 0-15 | 1729 |
|  | 15-30 | 1958 |
|  | 30-60 | 4110 |
|  | 60-90 | 4290 |
| **MS** | 0-15 | 1650 |
|  | 15-30 | 1943 |
|  | 30-60 | 4090 |
|  | 60-90 | 4240 |
| **MSW** | 0-15 | 1770 |
|  | 15-30 | 1949 |
|  | 30-60 | 4315 |
|  | 60-90 | 4246 |
| **MAAA** | 0-15 | 1742 |
|  | 15-30 | 1932 |
|  | 30-60 | 3962 |
|  | 60-90 | 4246 |
| **MOA** | 0-15 | 1783 |
|  | 15-30 | 2028 |
|  | 30-60 | 4053 |
|  | 60-90 | 4243 |

**^1^** Initial fixed depth increment

**Table S2. Typical (long-term) tillage practices at the Wisconsin Integrated Cropping Systems Trial.** Practices may vary slightly in any given year depending on soil conditions and weed pressure.

| **System** | **Phase** | **Spring 1°** | **Spring 2°** | **Spring 3°** | **Summer 1°** | **Summer 2°** | **Summer 3°** | **Fall 1°** | **Fall 2°** | **Fall 3°** |
| --- | --- | --- | --- | --- | --- | --- | --- | --- | --- | --- |
| **Maize** | Maize | -- | FC (1x) | -- | -- | -- | -- | DC (1x) | -- | -- |
| **MS** | Maize | -- | -- | -- | -- | -- | -- | -- | -- | -- |
|  | Soybean | -- | -- | -- | -- | -- | -- | -- | -- | ST (1x) |
| **MSW** | Maize | -- | FC (3x) | TW (2x)  RH (3x)  RC (2x)  DH (1x) | -- | -- | -- | DC (1x) | -- | -- |
|  | Soybean | -- | FC (3x) | TW (2x)  RH (3x)  RC (2x)  DH (1x) | -- | -- | -- | -- | FC (2x) | -- |
|  | Wheat | -- | -- | -- | DC (1x) | FC (1x) | -- | DC (2x) | -- | -- |
| **MAAA** | Maize | -- | FC (1x) | -- | -- | -- | -- | DC (1x) | -- | -- |
|  | *ds.* Alfalfa | -- | FC (2x) | -- | -- | -- | -- | -- | -- | -- |
|  | Alfalfa | -- | -- | -- | -- | -- | -- | -- | -- | -- |
|  | Alfalfa | -- | -- | -- | -- | -- | -- | DC (2x) | -- | -- |
| **MOA** | Maize | -- | FC (3x) | TW (2x)  RH (3x)  RC (2x)  DH (1x) | -- | -- | -- | DC (1x) | -- | -- |
|  | Oats/Alfalfa | -- | FC (2x) | -- | -- | -- | -- | -- | -- | -- |
|  | Alfalfa | -- | -- | -- | -- | -- | -- | DC (2x) | -- | -- |

Soils sampled in fall. Where applicable this is done after crop harvest but before tillage.

**Key:**

DC – disk chisel (~ 20 cm)

DH – disk hiller (~ 10 cm)

FC – field cultivator (~ 13 cm)

RH – rotary hoe (~ 2.5 cm)

ST – strip tillage (~ 10 cm)

TW – tine weeder (~ 2.5 cm)

**Table S3.** C concentration, bulk density, ESM stock and FD stock percent change from t0 to t1 for all treatments and depths

| Experiment | Treatment | ESM depth (cm) | C concentration average percent change (%) | BD average percent change (%) | ESM stock average percent change (%) | FD stock average percent change (%) |
| --- | --- | --- | --- | --- | --- | --- |
| Century Maize | CMT | 0-15 | 2.93 | -18.90 | 2.93 | -15.92 |
|  |  | 15-30 | 0.81 | -11.51 | 0.81 | -6.24 |
|  |  | 30-60 | -4.57 | -3.50 | -4.57 | -5.06 |
|  |  | 60-100 | -2.36 | -0.11 | -2.36 | -0.24 |
|  |  | 100-200 | -7.49 | -0.11 | -7.49 | -2.33 |
|  | CMT +WCC | 0-15 | 20.59 | -17.10 | 20.60 | 0.59 |
|  |  | 15-30 | 9.21 | -16.44 | 9.20 | -3.06 |
|  |  | 30-60 | -5.68 | -11.57 | -5.68 | -9.41 |
|  |  | 60-100 | -14.36 | 0.00 | -14.36 | -9.50 |
|  |  | 100-200 | -18.68 | -0.21 | -18.68 | -13.68 |
|  | OMT | 0-15 | 41.33 | -15.88 | 41.33 | 20.56 |
|  |  | 15-30 | 22.26 | -14.49 | 22.27 | 10.22 |
|  |  | 30-60 | 5.01 | 1.00 | 5.01 | 11.24 |
|  |  | 60-100 | 0.59 | 0.00 | 0.59 | 3.28 |
|  |  | 100-200 | 7.44 | -0.10 | 7.44 | 11.61 |
| Century Wheat | IW | 0-15 | 5.95 | -10.43 | 5.95 | -4.91 |
|  |  | 15-30 | 0.94 | -9.75 | 0.94 | -7.17 |
|  |  | 30-60 | 2.97 | 2.16 | 2.97 | 6.13 |
|  |  | 60-100 | -0.03 | -0.73 | -0.03 | 0.26 |
|  |  | 100-200 | -7.45 | -0.21 | -7.45 | -2.95 |
|  | IW + N | 0-15 | 9.51 | -16.69 | 9.51 | -9.02 |
|  |  | 15-30 | 6.47 | -5.57 | 6.47 | 2.53 |
|  |  | 30-60 | 7.35 | 6.03 | 7.35 | 16.40 |
|  |  | 60-100 | 15.74 | 1.11 | 15.74 | 17.78 |
|  |  | 100-200 | 9.39 | 0.00 | 9.39 | 11.94 |
|  | RW | 0-15 | -4.04 | -15.50 | -4.04 | -18.44 |
|  |  | 15-30 | 0.85 | -3.36 | 0.85 | -1.06 |
|  |  | 30-60 | -0.63 | 4.43 | -0.63 | 5.91 |
|  |  | 60-100 | -3.41 | 1.85 | -3.41 | -0.68 |
|  |  | 100-200 | -11.21 | -0.11 | -11.21 | -9.66 |
|  | RW + N | 0-15 | 3.81 | -18.15 | 3.81 | -14.24 |
|  |  | 15-30 | 0.02 | 0.78 | 0.02 | 2.25 |
|  |  | 30-60 | 3.28 | 4.20 | 3.28 | 8.91 |
|  |  | 60-100 | -3.24 | 0.11 | -3.24 | -2.29 |
|  |  | 100-200 | 5.81 | 0.11 | 5.81 | 3.27 |
|  | RW + WCC | 0-15 | 4.44 | -18.69 | 4.44 | -14.53 |
|  |  | 15-30 | 2.54 | -4.40 | 2.54 | 0.73 |
|  |  | 30-60 | -4.99 | 4.32 | -4.99 | 1.70 |
|  |  | 60-100 | 5.37 | -1.44 | 5.37 | 4.60 |
|  |  | 100-200 | -3.32 | -1.18 | -3.32 | 1.83 |
| WICST | Maize | 0-15 | -19.30 | 6.51 | -19.30 | -15.02 |
|  |  | 15-30 | -19.98 | 2.49 | -19.98 | -19.77 |
|  |  | 30-60 | -19.49 | 0.00 | -19.49 | -23.46 |
|  |  | 60-90 | -40.72 | 0.00 | -40.72 | -41.48 |
|  | MS | 0-15 | -4.69 | 19.24 | -4.69 | 12.24 |
|  |  | 15-30 | -11.45 | 5.53 | -11.45 | -12.22 |
|  |  | 30-60 | 3.01 | 0.00 | 3.01 | -9.20 |
|  |  | 60-90 | -25.30 | 0.00 | -25.30 | -22.50 |
|  | MSW | 0-15 | -11.84 | 10.02 | -11.84 | -4.20 |
|  |  | 15-30 | -14.32 | 4.48 | -14.32 | -13.56 |
|  |  | 30-60 | -10.11 | 0.00 | -10.11 | -16.32 |
|  |  | 60-90 | -30.19 | 0.00 | -30.19 | -30.39 |
|  | MAAA | 0-15 | -1.34 | 4.30 | -1.34 | 1.76 |
|  |  | 15-30 | -12.84 | -0.54 | -12.84 | -14.59 |
|  |  | 30-60 | -5.65 | 0.00 | -5.65 | -7.21 |
|  |  | 60-90 | -16.43 | 0.00 | -16.43 | -14.39 |
|  | MOA | 0-15 | -0.87 | 6.64 | -0.87 | 4.34 |
|  |  | 15-30 | -6.44 | -1.89 | -6.44 | -10.39 |
|  |  | 30-60 | -10.48 | 0.00 | -10.48 | -12.12 |
|  |  | 60-90 | -36.65 | 0.00 | -36.65 | -31.47 |

**Table S4.** T-test results with confidence intervals for the difference in mean between 1993 and 2012 Century maize bulk density measurements

| **Treatment** | **ESM depth (cm)** | **Difference in mean between 1993 and 2012 BD (g cm^-3^)** | **95% CI for difference in mean between 1993 and 2012 BD (g cm^-3^)** | **t-statistic** | **Hedges' g - effect size** | **p-value** |
| --- | --- | --- | --- | --- | --- | --- |
| **CMT** | 0-15 | -0.29 | -0.41, -0.16 | -5.75 | -3.06 | 0.002 |
|  | 15-30 | -0.17 | -0.39, 0.05 | -1.97 | -1.05 | 0.104 |
|  | 30-60 | -0.05 | -0.21, 0.11 | -0.80 | -0.42 | 0.454 |
|  | 60-100 | 0.00 | -0.11, 0.10 | -0.04 | -0.02 | 0.973 |
|  | 100-200 | 0.00 | -0.10, 0.10 | -0.04 | -0.02 | 0.970 |
| **CMT +WCC** | 0-15 | -0.25 | -0.36 , -0.14 | -5.12 | -2.73 | 0.001 |
|  | 15-30 | -0.24 | -0.41, -0.08 | -3.36 | -1.79 | 0.010 |
|  | 30-60 | -0.18 | -0.28, -0.08 | -4.05 | -2.16 | 0.002 |
|  | 60-100 | 0.00 | -0.11 , 0.11 | 0.00 | 0.00 | 1.000 |
|  | 100-200 | -0.003 | -0.11, 0.10 | -0.07 | -0.04 | 0.945 |
| **OMT** | 0-15 | -0.24 | -0.42, -0.06 | -3.38 | -1.80 | 0.018 |
|  | 15-30 | -0.22 | -0.30, -0.13 | -5.73 | -3.05 | 0.0003 |
|  | 30-60 | 0.01 | -0.11, 0.14 | 0.26 | 0.14 | 0.799 |
|  | 60-100 | 0.00 | -0.06 , 0.06 | 0.00 | 0.00 | 1.000 |
|  | 100-200 | 0.00 | -0.11 , 0.11 | -0.03 | -0.02 | 0.974 |

**Table S5.** T-test results with confidence intervals for the difference between 1993 and 2012 Century maize carbon concentrations

| **Treatment** | **ESM depth (cm)** | **Difference in mean between 1993 and 2012 C concentrations (g kg ^-1^)** | **95% CI for difference in mean between 1993 and 2012 C concentrations**  **(g kg ^-1^)** | **t-statistic** | **Hedges' g - effect size** | **p-value** |
| --- | --- | --- | --- | --- | --- | --- |
| **CMT** | 0-15 | 0.29 | -0.72, 1.30 | 0.66 | 0.35 | 0.529 |
|  | 15-30 | 0.07 | -1.05, 1.19 | 0.14 | 0.08 | 0.890 |
|  | 30-60 | -0.34 | -1.49, 0.82 | -0.67 | -0.36 | 0.522 |
|  | 60-100 | -0.15 | -1.27, 0.97 | -0.30 | -0.16 | 0.773 |
|  | 100-200 | -0.28 | -1.23, 0.66 | -0.67 | -0.36 | 0.518 |
| **CMT + WCC** | 0-15 | 1.91 | 0.63, 3.19 | 3.35 | 1.79 | 0.008 |
|  | 15-30 | 0.80 | -1.04, 2.64 | 0.99 | 0.53 | 0.348 |
|  | 30-60 | -0.42 | -2.49, 1.65 | -0.46 | -0.24 | 0.657 |
|  | 60-100 | -0.89 | -2.74, 0.95 | -1.08 | -0.58 | 0.306 |
|  | 100-200 | -0.76 | -2.26, 0.74 | -1.17 | -0.63 | 0.274 |
| **OMT** | 0-15 | 4.01 | 2.90, 5.12 | 8.09 | 4.31 | 0.00001 |
|  | 15-30 | 1.99 | 0.45, 3.52 | 2.92 | 1.56 | 0.017 |
|  | 30-60 | 0.39 | -0.78, 1.56 | 0.74 | 0.40 | 0.474 |
|  | 60-100 | 0.04 | -0.85, 0.93 | 0.09 | 0.05 | 0.926 |
|  | 100-200 | 0.26 | -0.52, 1.03 | 0.74 | 0.40 | 0.475 |

**Table S6.** Mean and standard deviation of Century maize carbon concentrations and bulk density by treatment and depth

| **Treatment** | **ESM depth (cm)** | | **1993 C concentration (g kg ^-1^)** | **2012 C concentration (g kg ^-1^)** | | **1993 bulk density (g cm ^-3^)** | **2012 bulk density (g cm ^-3^)** |
| --- | --- | --- | --- | --- | --- | --- | --- |
| **CMT** | 0-15 | 9.82 ± 0.94 | | | 10.10 ± 0.51 | 1.51 ± 0.03 | 1.22 ± 0.12 |
|  | 15-30 | 8.78 ± 0.82 | | | 8.85 ± 0.91 | 1.49 ± 0.03 | 1.32 ± 0.21 |
|  | 30-60 | 7.35 ± 1.06 | | | 7.02 ± 0.62 | 1.53 ± 0.06 | 1.47 ± 0.15 |
|  | 60-100 | 6.30 ± 0.91 | | | 6.15 ± 0.82 | 1.59 ± 0.08 | 1.58 ± 0.08 |
|  | 100-200 | 3.80 ± 0.68 | | | 3.51 ± 0.79 | 1.52 ± 0.07 | 1.52 ± 0.08 |
| **CMT + WCC** | 0-15 | 9.27 ± 0.85 | | | 11.18 ± 1.11 | 1.46 ± 0.09 | 1.21 ± 0.07 |
|  | 15-30 | 8.70 ± 1.10 | | | 9.50 ± 1.65 | 1.48 ± 0.09 | 1.24 ± 0.16 |
|  | 30-60 | 7.45 ± 1.43 | | | 7.03 ± 1.76 | 1.57 ± 0.08 | 1.39 ± 0.07 |
|  | 60-100 | 6.21 ± 1.29 | | | 5.32 ± 1.55 | 1.62 ± 0.09 | 1.62 ± 0.09 |
|  | 100-200 | 4.09 ± 1.38 | | | 3.33 ± 0.81 | 1.58 ± 0.08 | 1.57 ± 0.08 |
| **OMT** | 0-15 | 9.70 ± 0.90 | | | 13.71 ± 0.82 | 1.52 ± 0.03 | 1.28 ± 0.17 |
|  | 15-30 | 8.92 ± 1.00 | | | 10.90 ± 1.33 | 1.48 ± 0.05 | 1.27 ± 0.08 |
|  | 30-60 | 7.77 ± 0.86 | | | 8.16 ± 0.96 | 1.49 ± 0.08 | 1.51 ± 0.11 |
|  | 60-100 | 6.45 ± 0.70 | | | 6.48 ± 0.68 | 1.64 ± 0.05 | 1.64 ± 0.05 |
|  | 100-200 | 3.46 ± 0.62 | | | 3.71 ± 0.58 | 1.61 ± 0.09 | 1.61 ± 0.09 |

**Table S7.** T-test results with confidence intervals for the difference between 1993 and 2012 Century wheat bulk density measurements

| **Treatment** | **ESM depth (cm)** | **Difference in mean between 1993 and 2012 BD (g cm^-3^)** | **95% CI for difference in mean between 1993 and 2012 BD (g cm^-3^)** | **t-statistic** | **Hedges' g - effect size** | **p-value** |
| --- | --- | --- | --- | --- | --- | --- |
| IW | 0-15 | -0.15 | -0.26, -0.04 | -3.41 | -1.82 | 0.014 |
|  | 15-30 | -0.14 | -0.31, 0.02 | -2.21 | -1.18 | 0.075 |
|  | 30-60 | 0.03 | -0.09, 0.15 | 0.67 | 0.35 | 0.527 |
|  | 60-100 | -0.01 | -0.16, 0.14 | -0.18 | -0.09 | 0.865 |
|  | 100-200 | -0.003 | -0.11, 0.10 | -0.07 | -0.04 | 0.945 |
| IW + N | 0-15 | -0.25 | -0.42, -0.08 | -3.40 | -1.81 | 0.011 |
|  | 15-30 | -0.08 | -0.26, 0.10 | -1.10 | -0.59 | 0.313 |
|  | 30-60 | 0.09 | -0.04, 0.22 | 1.59 | 0.85 | 0.142 |
|  | 60-100 | 0.02 | -0.18, 0,21 | 0.19 | 0.10 | 0.853 |
|  | 100-200 | 0.00 | -0.12, 12 | 0.00 | 0.00 | 1.000 |
| RW | 0-15 | -0.23 | -0.36, -0.11 | -4.19 | -2.23 | 0.002 |
|  | 15-30 | -0.05 | -0.13, 0.03 | -1.37 | -0.73 | 0.200 |
|  | 30-60 | 0.07 | -0.03, 0.16 | 1.62 | 0.87 | 0.141 |
|  | 60-100 | 0.03 | -0.14, 0.19 | 0.39 | 0.21 | 0.707 |
|  | 100-200 | 0.00 | -0.11, 0.10 | -0.04 | -0.02 | 0.973 |
| RW + N | 0-15 | -0.27 | -0.39, -0.14 | -5.11 | -2.72 | 0.002 |
|  | 15-30 | 0.01 | -0.12, 0.14 | 0.22 | 0.12 | 0.834 |
|  | 30-60 | 0.06 | -0.05, 0.18 | 1.20 | 0.64 | 0.258 |
|  | 60-100 | 0.002 | -0.13, 0.14 | 0.03 | 0.01 | 0.979 |
|  | 100-200 | 0.002 | -0.14, 0.14 | 0.03 | 0.01 | 0.980 |
| RW + WCC | 0-15 | -0.27 | -0.37, 0.17 | -6.28 | -3.34 | 0.0001 |
|  | 15-30 | -0.06 | -0.22, 0.09 | -0.99 | -0.53 | 0.357 |
|  | 30-60 | 0.07 | -0.05, 0.18 | 1.30 | 0.69 | 0.225 |
|  | 60-100 | -0.02 | -0.19, 0.15 | -0.31 | -0.16 | 0.764 |
|  | 100-200 | -0.02 | -0.16, 0.13 | -0.28 | -0.15 | 0.785 |

**Table S8.** T-test results with confidence intervals for the difference between 1993 and 2012 Century wheat carbon concentrations

| **Treatment** | **ESM depth (cm)** | **Difference in mean between 1993 and 2012 C concentrations (g C kg ^-1^)** | **95% CI for difference in mean between 1993 and 2012 C concentrations (g C kg ^-1^)** | | **t-statistic** | **Hedges' g - effect size** | **p-value** |
| --- | --- | --- | --- | --- | --- | --- | --- |
| IW | 0-15 | 0.55 | -0.69, 1.80 | 1.03 | | 0.55 | 0.333 |
|  | 15-30 | 0.08 | -1.27, 1.43 | 0.13 | | 0.07 | 0.897 |
|  | 30-60 | 0.22 | -0.95, 1.38 | 0.42 | | 0.22 | 0.686 |
|  | 60-100 | -0.002 | -1.05, 1.04 | -0.004 | | -0.002 | 0.997 |
|  | 100-200 | -0.30 | -1.63, 1.02 | -0.54 | | -0.29 | 0.605 |
| IW + N | 0-15 | 0.82 | -0.89, 2.53 | 1.08 | | 0.58 | 0.307 |
|  | 15-30 | 0.49 | -1.77, 2.75 | 0.49 | | 0.26 | 0.636 |
|  | 30-60 | 0.45 | -2.08, 2.99 | 0.40 | | 0.21 | 0.697 |
|  | 60-100 | 0.86 | -1.27, 2.99 | 0.95 | | 0.51 | 0.373 |
|  | 100-200 | 0.43 | -1.37, 2.23 | 0.53 | | 0.28 | 0.609 |
| RW | 0-15 | -0.39 | -1.62, 0.84 | -0.72 | | -0.38 | 0.492 |
|  | 15-30 | 0.07 | -1.63, 1.77 | 0.09 | | 0.05 | 0.927 |
|  | 30-60 | -0.04 | -1.33, 1.24 | -0.08 | | -0.04 | 0.939 |
|  | 60-100 | -0.21 | -1.57, 1,15 | -0.34 | | -0.18 | 0.738 |
|  | 100-200 | -0.44 | -1.53, 0.64 | -0.91 | | -0.48 | 0.385 |
| RW + N | 0-15 | 0.38 | -0.73, 1,48 | 0.78 | | 0.42 | 0.455 |
|  | 15-30 | 0.002 | -1.37, 1.38 | 0.003 | | 0.002 | 0.998 |
|  | 30-60 | 0.25 | -1.10, 1.61 | 0.44 | | 0.23 | 0.675 |
|  | 60-100 | -0.22 | -1.94, 1.49 | -0.31 | | -0.16 | 0.768 |
|  | 100-200 | 0.24 | -1.23, 1.71 | 0.37 | | 0.19 | 0.722 |
| RW + WCC | 0-15 | 0.42 | -0.52, 1.36 | 1.00 | | 0.54 | 0.339 |
|  | 15-30 | 0.22 | -0.92, 1.36 | 0.43 | | 0.23 | 0.677 |
|  | 30-60 | -0.38 | -1.62, 0.85 | -0.69 | | -0.37 | 0.503 |
|  | 60-100 | 0.34 | -1.16, 1.84 | 0.50 | | 0.27 | 0.629 |
|  | 100-200 | -0.13 | -1.42, 1.16 | -0.22 | | -0.12 | 0.828 |

**Table S9.** Mean and standard deviation of Century wheat carbon concentrations and bulk density by treatment and depth

| **Treatment** | **ESM depth (cm)** | **1993 C concentration (g kg ^-1^)** | **2012 C concentration (g kg ^-1^)** | **1993 bulk density (g cm ^-3^)** | **2012 bulk density (g cm ^-3^)** |
| --- | --- | --- | --- | --- | --- |
| **IW** | 0-15 | 9.29 ± 0.60 | 9.84 ± 1.16 | 1.47 ± 0.03 | 1.32 ± 0.10 |
|  | 15-30 | 8.39 ± 0.83 | 8.47 ± 1.20 | 1.47 ± 0.03 | 1.33 ± 0.16 |
|  | 30-60 | 7.26 ± 0.74 | 7.47 ± 1.02 | 1.54 ± 0.05 | 1.57 ± 0.11 |
|  | 60-100 | 6.48 ± 0.71 | 6.48 ± 0.89 | 1.60 ± 0.11 | 1.59 ± 0.12 |
|  | 100-200 | 4.06 ± 1.25 | 3.75 ± 0.54 | 1.57 ± 0.08 | 1.57 ± 0.08 |
| **IW + N** | 0-15 | 8.60 ± 1.12 | 9.42 ± 1.48 | 1.49 ± 0.07 | 1.24 ± 0.16 |
|  | 15-30 | 7.60 ± 1.52 | 8.09 ± 1.94 | 1.47 ± 0.05 | 1.38 ± 0.17 |
|  | 30-60 | 6.18 ± 1.72 | 6.63 ± 2.17 | 1.49 ± 0.10 | 1.58 ± 0.10 |
|  | 60-100 | 5.45 ± 0.91 | 6.31 ± 2.01 | 1.50 ± 0.14 | 1.52 ± 0.16 |
|  | 100-200 | 4.54 ± 1.32 | 4.96 ± 1.47 | 1.56 ± 0.09 | 1.56 ± 0.10 |
| **RW** | 0-15 | 9.64 ± 1.09 | 9.25 ± 0.76 | 1.51 ± 0.08 | 1.27 ± 0.11 |
|  | 15-30 | 8.39 ± 1.22 | 8.46 ± 1.40 | 1.49 ± 0.06 | 1.44 ± 0.06 |
|  | 30-60 | 7.15 ± 1.13 | 7.11 ± 0.83 | 1.50 ± 0.08 | 1.57 ± 0.05 |
|  | 60-100 | 6.15 ± 1.06 | 5.94 ± 1.06 | 1.53 ± 0.15 | 1.56 ± 0.10 |
|  | 100-200 | 3.94 ± 0.80 | 3.50 ± 0.88 | 1.56 ± 0.08 | 1.56 ± 0.09 |
| **RW + N** | 0-15 | 9.86 ± 0.58 | 10.24 ± 1.02 | 1.48 ± 0.05 | 1.21 ± 0.12 |
|  | 15-30 | 9.07 ± 0.79 | 9.07 ± 1.25 | 1.49 ± 0.05 | 1.50 ± 0.12 |
|  | 30-60 | 7.72 ± 0.65 | 7.98 ± 1.26 | 1.51 ± 0.09 | 1.57 ± 0.10 |
|  | 60-100 | 6.94 ± 0.84 | 6.71 ± 1.59 | 1.53 ± 0.10 | 1.53 ± 0.11 |
|  | 100-200 | 4.15 ± 1.10 | 4.39 ± 1.18 | 1.59 ± 0.11 | 1.59 ± 0.11 |
| **RW + WCC** | 0-15 | 9.50 ± 0.78 | 9.92 ± 0.67 | 1.45 ± 0.07 | 1.18 ± 0.08 |
|  | 15-30 | 8.63 ± 0.86 | 8.85 ± 0.91 | 1.44 ± 0.06 | 1.38 ± 0.15 |
|  | 30-60 | 7.72 ± 0.94 | 7.34 ± 0.98 | 1.51 ± 0.07 | 1.57 ± 0.10 |
|  | 60-100 | 6.24 ± 1.10 | 6.58 ± 1.22 | 1.62 ± 0.13 | 1.59 ± 0.13 |
|  | 100-200 | 3.86 ± 1.11 | 3.73 ± 0.86 | 1.53 ± 0.11 | 1.51 ± 0.11 |

**Table S10.** T-test results with confidence intervals for the difference between 1989 and 2009 WICST bulk density measurements

| **Treatment** | **ESM depth (cm)** | **Difference in mean between 1989 and 2009 BD (g cm^-3^)** | **95% CI for difference in mean between 1989 and 2009 BD (g cm^-3^)** | **t-statistic** | **Hedges' g - effect size** | **p-value** |
| --- | --- | --- | --- | --- | --- | --- |
| **Maize** | 0-15 | 0.08 | 0.03, 0.12 | 3.78 | 1.49 | 0.001 |
|  | 15-30 | 0.03 | -0.02, 0.09 | 1.26 | 0.50 | 0.223 |
| **MS** | 0-15 | 0.21 | 0.14, 0.28 | 6.58 | 2.15 | 0.000001 |
|  | 15-30 | 0.07 | -0.01, 0.15 | 1.84 | 0.60 | 0.078 |
| **MSW** | 0-15 | 0.12 | 0.08, 0.15 | 6.60 | 1.60 | 0.00000001 |
|  | 15-30 | 0.06 | 0.03, 0.09 | 3.94 | 0.96 | 0.0004 |
| **MAAA** | 0-15 | 0.05 | 0.02, 0.08 | 3.19 | 0.71 | 0.002 |
|  | 15-30 | -0.01 | -0.03, 0.02 | -0.57 | -0.13 | 0.569 |
| **MOA** | 0-15 | 0.08 | 0.04, 0.011 | 4.48 | 1.20 | 0.00004 |
|  | 15-30 | -0.03 | -0.07, 0.02 | -1.04 | -0.28 | 0.304 |

*Note: 30-60 and 60-90 cm depths have been removed since bulk density values were assumed to stay the same from 1989 to 2009 below 30 cm.*

**Table S11.** T-test results with confidence intervals for the difference between 1989 and 2009 WICST carbon concentrations

| **Treatment** | **ESM depth (cm)** | **Difference in mean between 1989 and 2009 C concentrations (g C kg ^-1^)** | **95% CI for difference in mean between 1989 and 2009 C concentrations (g C kg ^-1^)** | **t-statistic** | **Hedges' g - effect size** | **p-value** |
| --- | --- | --- | --- | --- | --- | --- |
| **Maize** | 0-15 | -5.59 | -10.01, -1.16 | -2.62 | -1.03 | 0.016 |
|  | 15-30 | -4.36 | -10.53, 1.81 | -1.47 | -0.58 | 0.157 |
|  | 30-60 | -2.05 | -6.71, 2.61 | -0.91 | -0.36 | 0.371 |
|  | 60-90 | -2.87 | -6.11, 0.38 | -1.87 | -0.74 | 0.080 |
| **MS** | 0-15 | -1.19 | -5.24, 2.87 | -0.59 | -0.19 | 0.557 |
|  | 15-30 | -2.34 | -7.59, 2.91 | -0.91 | -0.30 | 0.372 |
|  | 30-60 | 0.28 | -3.76, 4.31 | 0.14 | 0.05 | 0.889 |
|  | 60-90 | -1.34 | -2.72, 0.05 | -1.96 | -0.64 | 0.058 |
| **MSW** | 0-15 | -3.10 | -5.72, -0.47 | -2.36 | -0.57 | 0.022 |
|  | 15-30 | -2.86 | -6.14, 0.42 | -1.74 | -0.42 | 0.087 |
|  | 30-60 | -0.95 | -3.58, 1.68 | -0.72 | -0.18 | 0.473 |
|  | 60-90 | -1.46 | -1.97, 0.96 | -5.86 | -1.43 | 0.0000004 |
| **MAAA** | 0-15 | -0.37 | -2.88, 2.13 | -0.30 | -0.07 | 0.767 |
|  | 15-30 | -2.90 | -6.31, 0.51 | -1.69 | -0.38 | 0.094 |
|  | 30-60 | -0.64 | -3.79, 2.51 | -0.40 | -0.09 | 0.688 |
|  | 60-90 | -0.87 | -1.69, -0.04 | -2.10 | -0.47 | 0.039 |
| **MOA** | 0-15 | -0.22 | -3.40, 2.96 | -0.14 | -0.04 | 0.890 |
|  | 15-30 | -1.26 | -5.31, 2.80 | -0.62 | -0.17 | 0.537 |
|  | 30-60 | -0.94 | -2.91, 1.03 | -0.96 | -0.26 | 0.344 |
|  | 60-90 | -2.59 | -4.50, -0.67 | -2.71 | -0.73 | 0.009 |

**Table S12.** Mean and standard deviation of WICST carbon concentrations and bulk density by treatment and depth

| **Treatment** | **ESM depth (cm)** | **1993 C concentration (g kg ^-1^)** | | **2012 C concentration (g kg ^-1^)** | | **1993 bulk density (g cm ^-3^)** | **2012 bulk density (g cm ^-3^)** |
| --- | --- | --- | --- | --- | --- | --- | --- |
| **IW** | 0-15 | 9.29 ± 0.60 | 9.84 ± 1.16 | | 1.47 ± 0.03 | | 1.32 ± 0.10 |
|  | 15-30 | 8.39 ± 0.83 | 8.47 ± 1.20 | | 1.47 ± 0.03 | | 1.33 ± 0.16 |
|  | 30-60 | 7.26 ± 0.74 | 7.47 ± 1.02 | | 1.54 ± 0.05 | | 1.57 ± 0.11 |
|  | 60-100 | 6.48 ± 0.71 | 6.48 ± 0.89 | | 1.60 ± 0.11 | | 1.59 ± 0.12 |
|  | 100-200 | 4.06 ± 1.25 | 3.75 ± 0.54 | | 1.57 ± 0.08 | | 1.57 ± 0.08 |
| **IW + N** | 0-15 | 8.60 ± 1.12 | 9.42 ± 1.48 | | 1.49 ± 0.07 | | 1.24 ± 0.16 |
|  | 15-30 | 7.60 ± 1.52 | 8.09 ± 1.94 | | 1.47 ± 0.05 | | 1.38 ± 0.17 |
|  | 30-60 | 6.18 ± 1.72 | 6.63 ± 2.17 | | 1.49 ± 0.10 | | 1.58 ± 0.10 |
|  | 60-100 | 5.45 ± 0.91 | 6.31 ± 2.01 | | 1.50 ± 0.14 | | 1.52 ± 0.16 |
|  | 100-200 | 4.54 ± 1.32 | 4.96 ± 1.47 | | 1.56 ± 0.09 | | 1.56 ± 0.10 |
| **RW** | 0-15 | 9.64 ± 1.09 | 9.25 ± 0.76 | | 1.51 ± 0.08 | | 1.27 ± 0.11 |
|  | 15-30 | 8.39 ± 1.22 | 8.46 ± 1.40 | | 1.49 ± 0.06 | | 1.44 ± 0.06 |
|  | 30-60 | 7.15 ± 1.13 | 7.11 ± 0.83 | | 1.50 ± 0.08 | | 1.57 ± 0.05 |
|  | 60-100 | 6.15 ± 1.06 | 5.94 ± 1.06 | | 1.53 ± 0.15 | | 1.56 ± 0.10 |
|  | 100-200 | 3.94 ± 0.80 | 3.50 ± 0.88 | | 1.56 ± 0.08 | | 1.56 ± 0.09 |
| **RW + N** | 0-15 | 9.86 ± 0.58 | 10.24 ± 1.02 | | 1.48 ± 0.05 | | 1.21 ± 0.12 |
|  | 15-30 | 9.07 ± 0.79 | 9.07 ± 1.25 | | 1.49 ± 0.05 | | 1.50 ± 0.12 |
|  | 30-60 | 7.72 ± 0.65 | 7.98 ± 1.26 | | 1.51 ± 0.09 | | 1.57 ± 0.10 |
|  | 60-100 | 6.94 ± 0.84 | 6.71 ± 1.59 | | 1.53 ± 0.10 | | 1.53 ± 0.11 |
|  | 100-200 | 4.15 ± 1.10 | 4.39 ± 1.18 | | 1.59 ± 0.11 | | 1.59 ± 0.11 |
| **RW + WCC** | 0-15 | 9.50 ± 0.78 | 9.92 ± 0.67 | | 1.45 ± 0.07 | | 1.18 ± 0.08 |
|  | 15-30 | 8.63 ± 0.86 | 8.85 ± 0.91 | | 1.44 ± 0.06 | | 1.38 ± 0.15 |
|  | 30-60 | 7.72 ± 0.94 | 7.34 ± 0.98 | | 1.51 ± 0.07 | | 1.57 ± 0.10 |
|  | 60-100 | 6.24 ± 1.10 | 6.58 ± 1.22 | | 1.62 ± 0.13 | | 1.59 ± 0.13 |
|  | 100-200 | 3.86 ± 1.11 | 3.73 ± 0.86 | | 1.53 ± 0.11 | | 1.51 ± 0.11 |

**Table S13.** T-test results with confidence intervals for the difference between 1993 and 2012 Century maize ESM stock estimates

| **Treatment** | **ESM depth (cm)** | **Difference in mean between 1993 and 2012 ESM stocks (Mg C ha ^-1^)** | **95% CI for difference in mean between 1993 and 2012 ESM stocks (Mg C ha ^-1^)** | **t-statistic** | **Hedges' g - effect size** | **p-value** |
| --- | --- | --- | --- | --- | --- | --- |
| **CMT** | 0-15 | 0.65 | -1.64, 2.94 | 0.66 | 0.35 | 0.529 |
|  | 15-30 | 0.16 | -2.35, 2.66 | 0.14 | 0.08 | 0.890 |
|  | 30-60 | -1.54 | -6.82, 3.75 | -0.67 | -0.36 | 0.522 |
|  | 60-100 | -0.94 | -8.02, 6.14 | -0.30 | -0.16 | 0.773 |
|  | 100-200 | -4.32 | -18.72, 10.08 | -0.67 | -0.36 | 0.518 |
| **CMT +WCC** | 0-15 | 4.18 | 1.38, 6.99 | 3.35 | 1.79 | 0.008 |
|  | 15-30 | 1.78 | -2.30, 5.86 | 0.99 | 0.53 | 0.348 |
|  | 30-60 | -1.99 | -11.75, 7.76 | -0.46 | -0.24 | 0.657 |
|  | 60-100 | -5.76 | -17.68, 6.16 | -1.08 | -0.58 | 0.306 |
|  | 100-200 | -12.04 | -35.66, 11.58 | -1.17 | -0.63 | 0.274 |
| **OMT** | 0-15 | 9.15 | 6.63, 11.68 | 8.09 | 4.31 | 0.00001 |
|  | 15-30 | 4.42 | 1.01, 7.82 | 2.92 | 1.56 | 0.017 |
|  | 30-60 | 1.74 | -3.49, 6.98 | 0.74 | 0.40 | 0.474 |
|  | 60-100 | 0.25 | -5.59, 6.09 | 0.09 | 0.05 | 0.926 |
|  | 100-200 | 4.13 | -8.28, 16.54 | 0.74 | 0.40 | 0.475 |

**Table S14.** T-test results with confidence intervals for the difference between 1993 and 2012 Century wheat ESM stock estimates

| **Treatment** | **ESM depth (cm)** | **Difference in mean between 1993 and 2012 ESM stocks (Mg C ha ^-1^)** | **95% CI for difference in mean between 1993 and 2012 ESM stocks (Mg C ha ^-1^)** | **t-statistic** | **Hedges' g - effect size** | **p-value** |
| --- | --- | --- | --- | --- | --- | --- |
| **IW** | 0-15 | 1.22 | -1.53, 3.97 | 1.03 | 0.55 | 0.333 |
|  | 15-30 | 0.17 | -2.80, 3.15 | 0.13 | 0.07 | 0.897 |
|  | 30-60 | 1.00 | -4.38, 6.37 | 0.42 | 0.22 | 0.686 |
|  | 60-100 | -0.01 | -6.71, 6.68 | -0.004 | 0.00 | 0.997 |
|  | 100-200 | -4.74 | -25.49, 16.02 | -0.54 | -0.29 | 0.605 |
| **IW + N** | 0-15 | 1.83 | -1.98, 5.64 | 1.08 | 0.58 | 0.307 |
|  | 15-30 | 1.08 | -3.88, 6.04 | 0.49 | 0.26 | 0.636 |
|  | 30-60 | 2.03 | -9.32, 13.39 | 0.40 | 0.21 | 0.697 |
|  | 60-100 | 5.14 | -7.64, 17.92 | 0.95 | 0.51 | 0.373 |
|  | 100-200 | 6.66 | -21.47, 34.80 | 0.53 | 0.28 | 0.609 |
| **RW** | 0-15 | -0.88 | -3.66, 1.90 | -0.72 | -0.38 | 0.492 |
|  | 15-30 | 0.16 | -3.64, 3.95 | 0.09 | 0.05 | 0.927 |
|  | 30-60 | -0.20 | -6.01, 5.61 | -0.08 | -0.04 | 0.939 |
|  | 60-100 | -1.29 | -9.62, 7.05 | -0.34 | -0.18 | 0.738 |
|  | 100-200 | -6.87 | -23.74, 10.00 | -0.91 | -0.48 | 0.385 |
| **RW + N** | 0-15 | 0.83 | -1.62, 3.29 | 0.78 | 0.42 | 0.455 |
|  | 15-30 | 0.00 | -3.07, 3.08 | 0.002 | 0.00 | 0.998 |
|  | 30-60 | 1.14 | -4.98, 7.27 | 0.44 | 0.23 | 0.675 |
|  | 60-100 | -1.38 | -11.86, 9.10 | -0.31 | -0.16 | 0.768 |
|  | 100-200 | 3.82 | -19.48, 27.12 | 0.37 | 0.19 | 0.722 |
| **RW + WCC** | 0-15 | 0.92 | -1.13, 2.96 | 1.00 | 0.54 | 0.339 |
|  | 15-30 | 0.47 | -1.99, 2.93 | 0.43 | 0.23 | 0.677 |
|  | 30-60 | -1.74 | -7.31, 3.84 | -0.69 | -0.37 | 0.503 |
|  | 60-100 | 2.17 | -7.53, 11.87 | 0.50 | 0.27 | 0.629 |
|  | 100-200 | -1.96 | -21.68, 17.77 | -0.22 | -0.12 | 0.828 |

**Table S15.** T-test results with confidence intervals for the difference between 1989 and 2009 WICST ESM stock estimates

| **Treatment** | **ESM depth (cm)** | **Difference in mean between 1989 and 2009 ESM stocks (Mg C ha ^-1^)** | **95% CI for difference in mean between 1989 and 2009 ESM stocks (Mg C ha ^-1^)** | **t-statistic** | **Hedges' g - effect size** | **p-value** |
| --- | --- | --- | --- | --- | --- | --- |
| **Maize** | 0-15 | -9.66 | -17.31, -2.01 | -2.62 | -1.03 | 0.016 |
|  | 15-30 | -8.53 | -20.60, 3.54 | -1.47 | -0.58 | 0.157 |
|  | 30-60 | -8.42 | -27.56, 10.72 | -0.91 | -0.36 | 0.371 |
|  | 60-90 | -12.29 | -26.22, 1.64 | -1.87 | -0.74 | 0.080 |
| **MS** | 0-15 | -1.96 | -8.65, 4.74 | -0.59 | -0.19 | 0.557 |
|  | 15-30 | -4.55 | -14.75, 5.66 | -0.91 | -0.30 | 0.372 |
|  | 30-60 | 1.14 | -15.36, 17.64 | 0.14 | 0.05 | 0.889 |
|  | 60-90 | -5.67 | -11.53, 0.20 | -1.96 | -0.64 | 0.058 |
| **MSW** | 0-15 | -5.48 | -10.13, -0.84 | -2.36 | -0.57 | 0.022 |
|  | 15-30 | -5.57 | -11.97, 0.82 | -1.74 | -0.42 | 0.087 |
|  | 30-60 | -4.10 | -15.45, 7.26 | -0.72 | -0.18 | 0.473 |
|  | 60-90 | -6.22 | -8.35, -4.09 | -5.86 | -1.43 | 0.0000004 |
| **MAAA** | 0-15 | -0.65 | -5.02, 3.71 | -0.30 | -0.07 | 0.767 |
|  | 15-30 | -5.60 | -12.19, 0.98 | -1.69 | -0.38 | 0.094 |
|  | 30-60 | -2.52 | -15.00, 9.96 | -0.40 | -0.09 | 0.688 |
|  | 60-90 | -3.68 | -7.17, -0.19 | -2.10 | -0.47 | 0.039 |
| **MOA** | 0-15 | -0.39 | -6.06, 5.28 | -0.14 | -0.04 | 0.890 |
|  | 15-30 | -2.55 | -10.78, 5.68 | -0.62 | -0.17 | 0.537 |
|  | 30-60 | -3.80 | -11.79, 4.19 | -0.96 | -0.26 | 0.344 |
|  | 60-90 | -10.97 | -19.12, 2,82 | -2.71 | -0.73 | 0.009 |
